# Astaxanthin promotes locomotor function recovery and attenuates tissue damage in rats following spinal cord injury: a systematic review and trial sequential analysis

**DOI:** 10.3389/fnins.2023.1255755

**Published:** 2023-10-10

**Authors:** Long-yun Zhou, Zi-ming Wu, Xu-qing Chen, Bin-bin Yu, Meng-xiao Pan, Lu Fang, Jian Li, Xue-jun Cui, Min Yao, Xiao Lu

**Affiliations:** ^1^Department of Rehabilitation Medicine, The First Affiliated Hospital of Nanjing Medical University, Nanjing, Jiangsu, China; ^2^Spine Disease Institute, Longhua Hospital, Shanghai University of Traditional Chinese Medicine, Shanghai, China; ^3^Key Laboratory of Theory and Therapy of Muscles and Bones, Ministry of Education, Shanghai University of Traditional Chinese Medicine, Shanghai, China; ^4^Department of Otolaryngology, Jiangsu Province Hospital of Chinese Medicine, Affiliated Hospital of Nanjing University of Chinese Medicine, Nanjing, Jiangsu, China

**Keywords:** astaxanthin, spinal cord injury, systematic review, trial sequential analysis, locomotor recovery, safety, neuroprotective mechanism, clinical translation

## Abstract

Spinal cord injury (SCI) is a catastrophic condition with few therapeutic options. Astaxanthin (AST), a natural nutritional supplement with powerful antioxidant activities, is finding its new application in the field of SCI. Here, we performed a systematic review to assess the neurological roles of AST in rats following SCI, and assessed the potential for clinical translation. Searches were conducted on PubMed, Embase, Cochrane Library, the Web of Science, China National Knowledge Infrastructure, WanFang data, Vip Journal Integration Platform, and SinoMed databases. Animal studies that evaluated the neurobiological roles of AST in a rat model of SCI were included. A total of 10 articles were included; most of them had moderate-to-high methodological quality, while the overall quality of evidence was not high. Generally, the meta-analyses revealed that rats treated with AST exhibited an increased Basso, Beattie, and Bresnahan (BBB) score compared with the controls, and the weighted mean differences (WMDs) between those two groups showed a gradual upward trend from days 7 (six studies, n = 88, WMD = 2.85, 95% CI = 1.83 to 3.87, *p* < 0.00001) to days 28 (five studies, n = 76, WMD = 6.42, 95% CI = 4.29 to 8.55, *p* < 0.00001) after treatment. AST treatment was associated with improved outcomes in spared white matter area, motor neuron survival, and SOD and MDA levels. Subgroup analyses indicated there were differences in the improvement of BBB scores between distinct injury types. The trial sequential analysis then firmly proved that AST could facilitate the locomotor recovery of rats following SCI. In addition, this review suggested that AST could modulate oxidative stress, neuroinflammation, neuron loss, and autophagy *via* multiple signaling pathways for treating SCI. Collectively, with a protective effect, good safety, and a systematic action mechanism, AST is a promising candidate for future clinical trials of SCI. Nonetheless, in light of the limitations of the included studies, larger and high-quality studies are needed for verification.

## Introduction

Spinal cord injury (SCI) is one of the most devastating events, afflicting thousands of human beings. Most SCIs are originated from traumatic injury. In China, it is estimated that there are 759,302 prevalent patients with traumatic SCI in total (Jiang et al., 2022). Globally, approximately 27.04 million people are affected by SCI, with over 700,000 new events of traumatic SCI annually (Gbd, 2019; Lucchesi et al., 2019). However, there are still few effective therapeutic strategies available.

The pathological mechanisms of SCI are complicated. Tissue structure and homeostasis are broken down instantly following the primary insult induced by trauma or ischemia. Subsequently, the secondary damage phase, involving oxidative stress, dysregulation of ion flow, neurotransmitter toxicity, immune cell activation, and neuronal loss, is gradually triggered (Liu et al., 2021; Salvador and Kipnis, 2022; Scheijen et al., 2022). Among these changes, oxidative stress is demonstrated to be triggered in just a few hours after initial injury (Liu et al., 2004; Tran et al., 2018). The free radicals derived from oxidative stress can further induce ionic dysregulation, endoplasmic reticulum stress, or caspases activation to cause neuronal damage, and activate glial cells to enlarge neuroinflammation response through a wide range of mechanisms (Anjum et al., 2020; Zrzavy et al., 2021; Ji et al., 2022). Thus, oxidative stress critically impacts the multifactorial pathological progression of SCI.

Astaxanthin (AST) is a carotenoid widely distributed in marine animals and can be synthesized by algae or bacteria, standing out among its chemical family for its powerful antioxidant activities (Abdol et al., 2022; Alugoju et al., 2022). Partly benefiting from its excellent antioxidant properties, AST exhibits roles against inflammation, apoptosis, and aging, and has shown promise in treating cardiovascular diseases, Alzheimer’s disease, Parkinson’s disease, and cancer in humans and animals (Chao et al., 2020; Donoso et al., 2021). With good safety and satisfactory bioactivities, AST has been approved by the Food and Drug Administration as a food supplement. Given the critical role of oxidative stress in SCI, AST is finding new application scope in the field of this disease (Abbaszadeh et al., 2022). Series of experimental studies have shown favorable efficacy for AST in the treatment of SCI (Masoudi et al., 2021; Abbaszadeh et al., 2022). However, the integrated pre-clinical evidence concerning the neuroprotective effects of AST on SCI are still absent. Furthermore, a review of the safety and action mechanism of AST remains yet to be performed. All of these parameters are important for clinical translation. Herein, we performed a comprehensive review to evaluate the neurobiological roles of AST for treating SCI in rats, and describe the potential for future clinical trials and applications.

## Materials and methods

This systematic review was presented according to the Preferred Reporting Items for Systematic reviews and Meta-Analyses (PRISMA) statement. No pre-registration was performed for this study. The design of this study followed the methods of previous studies (Yao et al., 2015; Sng et al., 2022; Zhou et al., 2022).

### Literature search

A systematic literature search was performed in the PubMed, Embase, Web of Science, Cochrane Library, China National Knowledge Infrastructure, WanFang data, Vip Journal Integration Platform, and SinoMed databases from their inception date to May 2023 (Sng et al., 2022; Zhou et al., 2022). Key words included “spinal cord injuries,” “spinal cord injury,” “spinal cord contusion,” “spinal cord compression,” “spinal cord trauma,” “astaxanthine,” “astaxanthin,” “E-astaxanthin’, “rats,” “rat,” and “murinae.” The bibliographies of the included studies were also screened to identify any additional relevant studies.

### Study selection

Two reviewers independently screened the title, abstracts, and full texts of the retrieved articles. Discrepancies were be resolved by consensus after discussion with a third reviewer. Studies that fulfilled all of the following criteria were included.

### Eligibility criteria

#### Types of studies

Studies assessing the neurobiological effects of AST on rats subjected to SCI were searched. Clinical case reports or only *in vitro* studies were excluded. No restrictions regarding language, publication date, or publication status were imposed (Sng et al., 2022).

#### Types of participants

There were no restrictions with respect to the age, sex, or strain of laboratory rats. The traumatic SCI model induced by contusion and compression injury were included. The establishment of rat models of SCI using non-traumatic ischemia, laceration, transection, genetic modification, or traumatic root avulsion was excluded (Yao et al., 2015).

#### Types of interventions

Studies assessing any type of AST compared with placebo control were included. The dosage, formulation, and administration methods of AST were unrestricted. Placebo control included saline, vehicle, and no treatment. Multiple treatment combinations (e.g., AST plus stem cell transplantation) were excluded (Sng et al., 2022).

#### Types of outcome measures

The 21-point Basso, Beattie, and Bresnahan (BBB) locomotor rating scale was considered as the primary outcome. This scale is a well-documented tool widely applied in evaluating the locomotor function recovery of rats following SCI (Basso et al., 1995). To reduce the risk of data heterogeneity, only data at the same time points were used in the analyses of BBB scores and the following outcomes (Zhou et al., 2022).

The secondary outcome measures included spared white matter area, the number of ventral horn motor neurons, malondialdehyde (MDA), and superoxide dismutase (SOD). Spared white matter area and the number of ventral horn motor neurons are widely applied indexes reflecting injury severity after SCI (Matsuda et al., 2020). MDA is the metabolic end product of lipid peroxide and a biomarker of cell oxidative damage, whereas SOD is a major enzyme in the antioxidant defense system and can limit further damage caused by reactive oxygen species (ROS) elements (Yin et al., 2019).

### Data extraction

Two investigators independently extracted the information of the included studies. The following data was extracted: the name of the first author, publication year, animal strain and gender, animal age and weight, number of animals per group, method used to induce SCI, injury level, AST administration (including dosage, method, timing, and times), and measured outcomes. In studies with multiple intervention arms, only data from the AST and negative control groups were extracted for analysis. If the data were represented as a graph without a numerical value, GetData Graph Digitizer 2.26^1^ was used to interpret graph data (Tian et al., 2020). If data were missing, we would contact the original authors and await a response for two weeks. In cases where there was no response, the related data would be included for qualitative analyses only.

### Risk of bias assessment

The methodological quality of studies was evaluated using the systematic Review Center for Laboratory animal Experimentation’s Risk of Bias (SYRCLE’s RoB) tool (Hooijmans et al., 2014). This tool includes 10 items for the assessment of selection bias, performance bias, detection bias, attrition bias, reporting bias, and other biases. The items were assessed with a judgment of “yes,” “no,” or “unclear,” indicating a low risk, a high risk, or that the risk of bias was not clear, respectively.

### Assessment of evidence quality

The quality of evidence in the included studies was evaluated according to the previously described approach (Charalambous et al., 2014, 2016). In this tool, the evidence quality of experimental laboratory animal studies (ELAS) was graded at three levels. The blinded randomized ELAS (bRELAS) and non-blinded RELAS (nbRELAS) were considered to provide higher quality evidence, followed by non-randomized RELAS (nRELAS) and uncontrolled ELAS (UELAS), then case series and reports. Subsequently, for correcting the confidence rating for single studies in each group, three domains were assessed for indicating the strengths or weaknesses of each study, involving: (1) sample sizes; (2) subject enrolment quality; and (3) the overall risk of bias according to SYRCLE’s RoB tool. Collectively, bRELAS with large sample sizes, rigorous criteria in subject enrolment, and low overall risk of bias were considered to produce the highest quality of evidence.

### Statistical analyses

Data from included studies were summarized and analyzed by the RevMan 5.3 software (provided by the Cochrane Collaboration). Two-sided *p* values of less than 0.05 were considered statistically significant. The mean, standard deviation, and the sample size of animals in each group were extracted for comparisons. In accordance with the recommendations of the Cochrane Handbook for Systematic Reviews of Interventions, for studies with multiple intervention groups, those groups were combined to enable a single pair-wise comparison (Higgins and Green, 2011). For outcomes with the same unit, the results were described as the weighted mean difference (WMD); otherwise, standardized MD (SMD) was employed. The 95% confidence intervals (CIs) were calculated for both types of outcomes. Heterogeneity between studies was evaluated by *p* value in the chi-squared test and Cochrane’s I^2^ (Higgins and Green, 2011). Heterogeneity is presumed in the event that the *p* value is less than 0.10, and is considered to be high when the I^2^ value is more than 50%. The random effects model was used because it provides the expected average of all samples of individual true effect sizes. Linear graphs were constructed by GraphPad Prism (version 5.01) software to highlight the dynamic WMDs of BBB scores and dynamic BBB score improvements in both groups.

Repeated updates (sequential multiplicity) and sparse data increase the risk of random error. The trial sequential analysis (TSA) adjusts the random-error risk and provides a conservative estimation for the required number of study subjects in order to carry out a definitive meta-analysis. Thus, we also conducted the trial sequential analysis method based on the primary outcome to confirm the results of meta-analyses. TSA 0.9.5.10 beta software (downloaded from https://ctu.dk/tsa/downloads) was used to construct a cumulative Z-curve by including each study according to the order of their publication year, and to estimate the required information size. A firm conclusion could be drawn without further research in cases where the cumulative Z-curve either crossed the monitoring boundary or required information size. The TSA was performed with a desired power of 90% and an alpha error of 5%.

## Results

### Study selection

**Figure 1:**
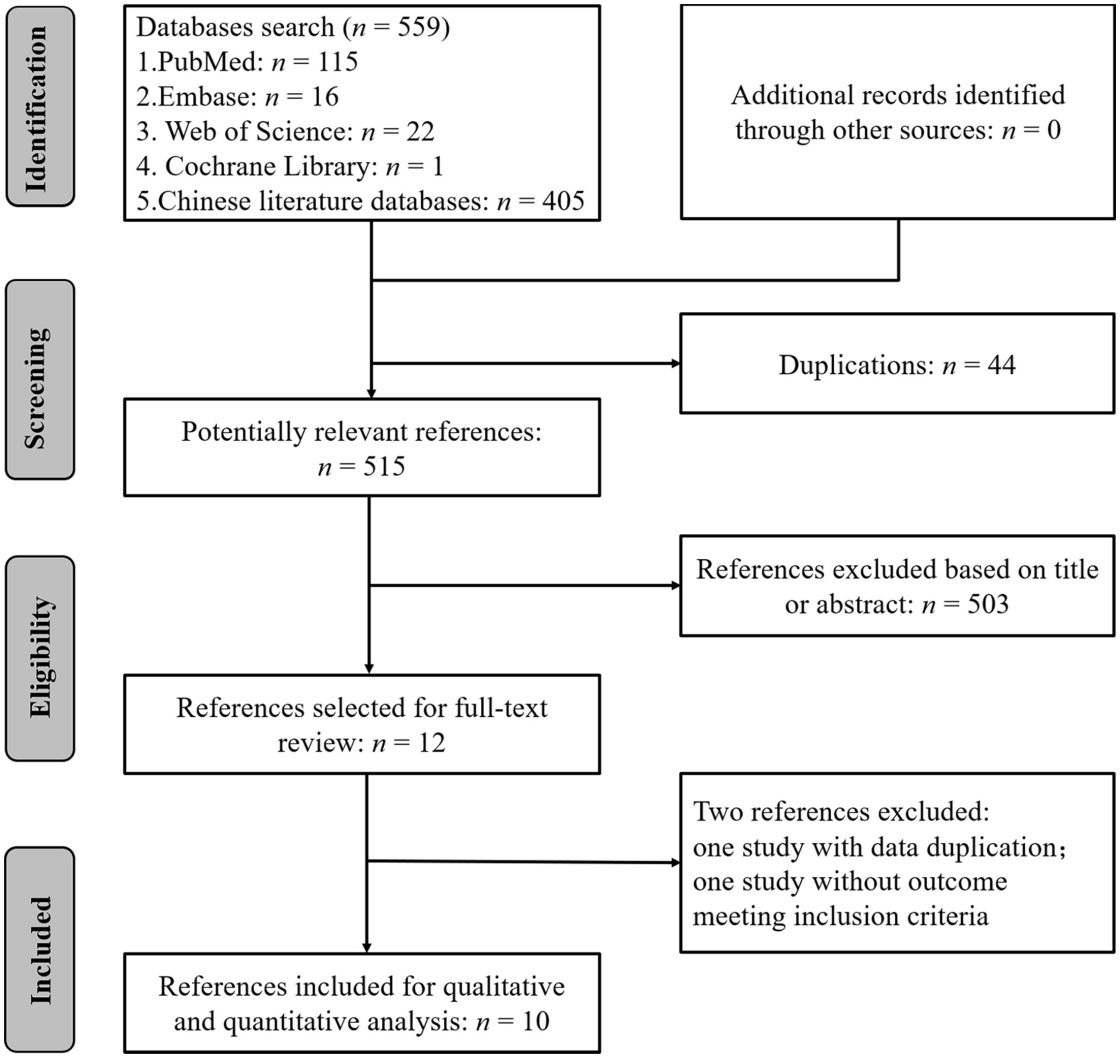
Summary of the literature identification and selection process.

Among the 559 studies identified with the search strategy, 44 duplicates were eliminated and another 503 studies were excluded by performing title and abstract screening. Of the 12 papers selected for full-text screening, one reference was excluded due to data duplication (Ren et al., 2018), and another one did not report an outcome that met the inclusion criteria (Fakhri et al., 2022). Ten studies were, therefore, included in this systematic review and meta-analysis (Figure 1) (Chen, 2014; Masoudi et al., 2017; Fakhri et al., 2018, 2019; Ren et al., 2019; Mohaghegh et al., 2020; Li et al., 2021; Masoudi et al., 2021; Abbaszadeh et al., 2022, 2023).

### Characteristics of the included studies

Of the 10 publications, eight articles were presented in English and the remaining two in Chinese (Chen, 2014; Ren et al., 2019). Seven of them used Wistar rats; the other three selected Sprague–Dawley rats (Chen, 2014; Ren et al., 2019; Li et al., 2021). Six studies induced SCI using a weight-drop impactor, and the remaining four used an aneurysm clip compression method (Fakhri et al., 2018, 2019; Abbaszadeh et al., 2022, 2023). All studies established SCI at the T8–T12 level. Administration of AST by intrathecal injection was employed in seven studies, with a dose of 0.005 mg/kg. The other studies used the intragastrical administration route, with an intervention dose ranging from 35 to 75 mg/kg (Chen, 2014; Ren et al., 2019; Li et al., 2021). All studies administrated AST immediately post injury, and most studies except three (Chen, 2014; Ren et al., 2019; Li et al., 2021) employed a single dose of AST (Table 1).

**Table 1:** Characteristics of included studies.

Study	Animals	Injury model	Animal number	Astaxanthin source	Groups	Administration time; treatment duration	Outcome
Abbaszadeh et al. (2023)	108 male Wistar rats (230-250 g)	T8-10 aneurysm clip compression 90 g*1 min	36/36/36	N/A	A: Sham SCI + saline B: SCI + 5% DMSO C: SCI + astaxanthin (0.005 mg/kg i.t.)	30 min after injury; once	Behavioral: BBB scale, beam walking test Other: MRI, BSCB permeability, spinal cord tissue edema, Western blot
Abbaszadeh et al. (2022)	90 male Wistar rats (230-250 g)	T8-10 aneurysm clip compression 90 g*1 min	30/30/30	N/A	A: Sham SCI + saline B: SCI + 5% DMSO C: SCI + astaxanthin (0.005 mg/kg i.t.)	30 min after injury; once	Behavioral: Combined behavioral score Histopathology: H&E staining, Fluoro-Jade B staining Other: SOD, MDA, glutathione peroxidase, total antioxidant capacity, Western blot
Masoudi et al. (2021)	96 adult male Wistar rats (2–3 months; 240–280 g)	T8-9 weight-drop impactor 10 g*50 mm	24/24/24/24	Sigma	A: Sham SCI B: SCI C: SCI + 5% DMSO D: SCI + astaxanthin (0.005 mg/kg i.t.)	30 min after injury; once	Behavioral: Von Frey test Histopathology: Nissl staining, LFB staining Other: SOD, MDA, glutathione peroxidase, catalase, Western blot
Li et al. (2021)	144 male and female rats (280 ± 20 g)	T9-10 weight-drop impactor 5 g*100 mm	48/48/48	Sigma	A: sham SCI + olive oil B: SCI + olive oil C: SCI + astaxanthin (75 mg/kg i.g.)	immediately; twice daily until sacrifice	Behavioral: BBB scale, Histopathology: H&E staining, TUNEL, immunofluorescence Other: SOD, MDA, spinal cord tissue edema, transmission electron microscope
Mohaghegh et al. (2020)	90 adult male Wistar rats (250-300 g)	T8-9 weight-drop impactor 10 g*50 mm	18/18/18/18/18	N/A	A: Sham SCI B: SCI + 5% DMSO C: SCI + astaxanthin (0.005 mg/kg i.t.) D: SCI + EPI-NCSCs E: SCI + astaxanthin + EPI-NCSCs	30 min after injury; once	Behavioral: BBB scale Histopathology: Nissl staining, LFB staining Other: PCR
Fakhri et al. (2019)	81 adult male Wistar rats (2–3 months, 230–260 g)	T8-9 aneurysm clip compression 90 g*1 min	27/27/27	Sigma	A: Sham SCI B: SCI + 5% DMSO C: SCI + astaxanthin (0.005 mg/kg i.t.)	30 min after injury; once	Behavioral: BBB scale, von Frey test, hotplate test, open field test Histopathology: Nissl staining, LFB staining Other: Western blot, body weight, auricle temperature, blood glucose
Ren et al. (2019)	90 adult male and female rats (280 ± 20 g)	T9-11 weight-drop impactor 5 g*100 mm	30/30/30	Sigma	A: sham SCI + olive oil B: SCI + olive oil C: SCI + astaxanthin (75 mg/kg i.g.)	immediately; twice daily until sacrifice	Behavioral: BBB scale Histopathology: Immunohistochemistry Other: Myeloperoxidase, IL-1β, IL-6, TNF-α, spinal cord tissue edema, transmission electron microscope
Fakhri et al. (2018)	75 adult male Wistar rats (230–270 g)	T8-9 aneurysm clip compression 90 g*1 min	25/25/25	Sigma	A: Sham SCI B: SCI + 5% DMSO C: SCI + astaxanthin (0.005 mg/kg i.t.)	30 min after injury; once	Behavioral: Inclined plane test, acetone drop test Histopathology: Nissl staining, LFB staining Other: Western blot
Masoudi et al. (2017)	108 adult male Wistar rats (2–3 months, 250–280 g)	T8-9 weight-drop impactor 10 g*50 mm	27/27/27/27	Sigma	A: Sham SCI B: SCI + 5% DMSO C: SCI + astaxanthin (0.005 mg/kg i.t.)	30 min after injury; once	Behavioral: BBB scale, Histopathology: Nissl staining, LFB staining Other: Western blot
Chen (2014)	66 female Sprague–Dawley rats (260-300 g)	T12 weight-drop impactor 10 g*60 mm	6/30/30	N/A	A: sham SCI B: SCI + vegetable oil C: SCI + astaxanthin (35 mg/kg i.g.)	5 min after injury; twice daily until sacrifice	Behavioral: BBB scale Histopathology: Nissl staining, immunohistochemistry Other: SOD, MDA

BBB, Basso, Beattie, and Bresnahan locomotor rating scale; BCSB, blood–spinal cord barrier; DMSO, dimethyl sulfoxide; EPI-NCSCs, epidermal neural crest stem cells; H&E, hematoxylin and eosin; i.g., intragastrical; i.t., intrathecal; LFB, luxol fast blue; MDA, malondialdehyde; MRI, magnetic resonance imaging; N/A, not available; PCR, polymerase chain reaction; SCI, spinal cord injury; SOD, superoxide dismutase; TUNEL, terminal dexynucleotidyl transferase mediated dUTP nick end labeling.

### Risk of bias within studies

According to the SYRCLE’s RoB tool, the mean number of reported items in the included studies was 4.1. Four studies adequately reported 50% of the details in the checklist (Masoudi et al., 2017; Fakhri et al., 2018; Ren et al., 2019; Li et al., 2021). All the included studies well described the items of “baseline characteristics” and “selective outcome reporting.” Six studies reported a design of outcome assessor blinding (Masoudi et al., 2017; Fakhri et al., 2018, 2019; Ren et al., 2019; Mohaghegh et al., 2020; Li et al., 2021), and three studies well addressed the item of incomplete outcome data (Masoudi et al., 2017; Fakhri et al., 2018; Masoudi et al., 2021). However, none or few studies reported the details of “sequence generation,” “allocation concealment,” “random housing,” “investigator blinding,” and “random outcome assessment.” Additionally, no other sources of bias such as the pooling of drugs, dropouts, unit of analysis errors, or design-specific bias were identified in the included studies (Table 2).

**Table 2:** Summary of risk of bias.

Study	1	2	3	4	5	6	7	8	9	10
Abbaszadeh et al. (2023)	Unclear	Yes	Unclear	Unclear	Unclear	Unclear	Unclear	Unclear	Yes	Yes
Abbaszadeh et al. (2022)	Unclear	Yes	Unclear	Unclear	Unclear	Unclear	Unclear	Unclear	Yes	Yes
Masoudi et al. (2021)	Unclear	Yes	Unclear	Unclear	Unclear	Unclear	Unclear	Yes	Yes	Yes
Li et al. (2021)	Yes	Yes	Unclear	Unclear	Unclear	Unclear	Yes	Unclear	Yes	Yes
Mohaghegh et al. (2020)	Unclear	Yes	Unclear	Unclear	Unclear	Unclear	Yes	Unclear	Yes	Yes
Fakhri et al. (2019)	Unclear	Yes	Unclear	Unclear	Unclear	Unclear	Yes	Unclear	Yes	Yes
Ren et al. (2019)	Yes	Yes	Unclear	Unclear	Unclear	Unclear	Yes	Unclear	Yes	Yes
Fakhri et al. (2018)	Unclear	Yes	Unclear	Unclear	Unclear	Unclear	Yes	Yes	Yes	Yes
Masoudi et al. (2017)	Unclear	Yes	Unclear	Unclear	Unclear	Unclear	Yes	Yes	Yes	Yes
Chen (2014)	Unclear	Yes	Unclear	Unclear	Unclear	Unclear	Unclear	Unclear	Yes	Yes

“Yes” indicates a low risk of bias; “No” indicates a high risk of bias; “Unclear” represents an unclear risk of bias. This tool includes 10 questions: (1) sequence generation; (2) baseline characteristics; (3) allocation concealment; (4) random housing; (5) investigator blinding; (6) random outcome assessment; (7) outcome assessor blinding; (8) incomplete outcome data; (9) selective outcome reporting; and (10) other sources of bias.

### Quality of evidence in studies

Overall, the evidence quality of the included studies was not high. Although all studies belonged to bRELAS or nbRELAS, three of them reported a design with a small sample size (Chen, 2014; Ren et al., 2019; Li et al., 2021) and another four did not adequately address the details of the subject enrolment quality (Fakhri et al., 2018, 2019; Abbaszadeh et al., 2022, 2023). Moreover, based on SYRCLE’s RoB tool, a potential risk of bias was widely presented in the included studies (Table 3).

**Table 3:** Summaries of the quality of evidence of the included studies.

Study	Study design	Study group sizes	Subject enrolment quality	Overall risk of bias
Abbaszadeh et al. (2023)	nbRELAS	Moderate	Unclear	Moderate/High
Abbaszadeh et al. (2022)	nbRELAS	Moderate	Unclear	Moderate/High
Masoudi et al. (2021)	nbRELAS	Moderate	Fairly	Moderate
Li et al. (2021)	bRELAS	Small	Fairly	Low/Moderate
Mohaghegh et al. (2020)	bRELAS	Moderate	Fairly	Moderate
Fakhri et al. (2019)	bRELAS	Moderate	Unclear	Moderate
Ren et al. (2019)	bRELAS	Small	Fairly	Low/Moderate
Fakhri et al. (2018)	bRELAS	Moderate	Unclear	Low/Moderate
Masoudi et al. (2017)	bRELAS	Moderate	Fairly	Low/Moderate
Chen (2014)	nbRELAS	Small	Fairly	Moderate/High

bRELAS, blinded randomized experimental laboratory animal studies; nbRELAS, non-blinded randomized experimental laboratory animal studies; nRELAS, non-randomized experimental laboratory animal studies.

### Effects of AST on locomotor recovery

**Figure 2:**
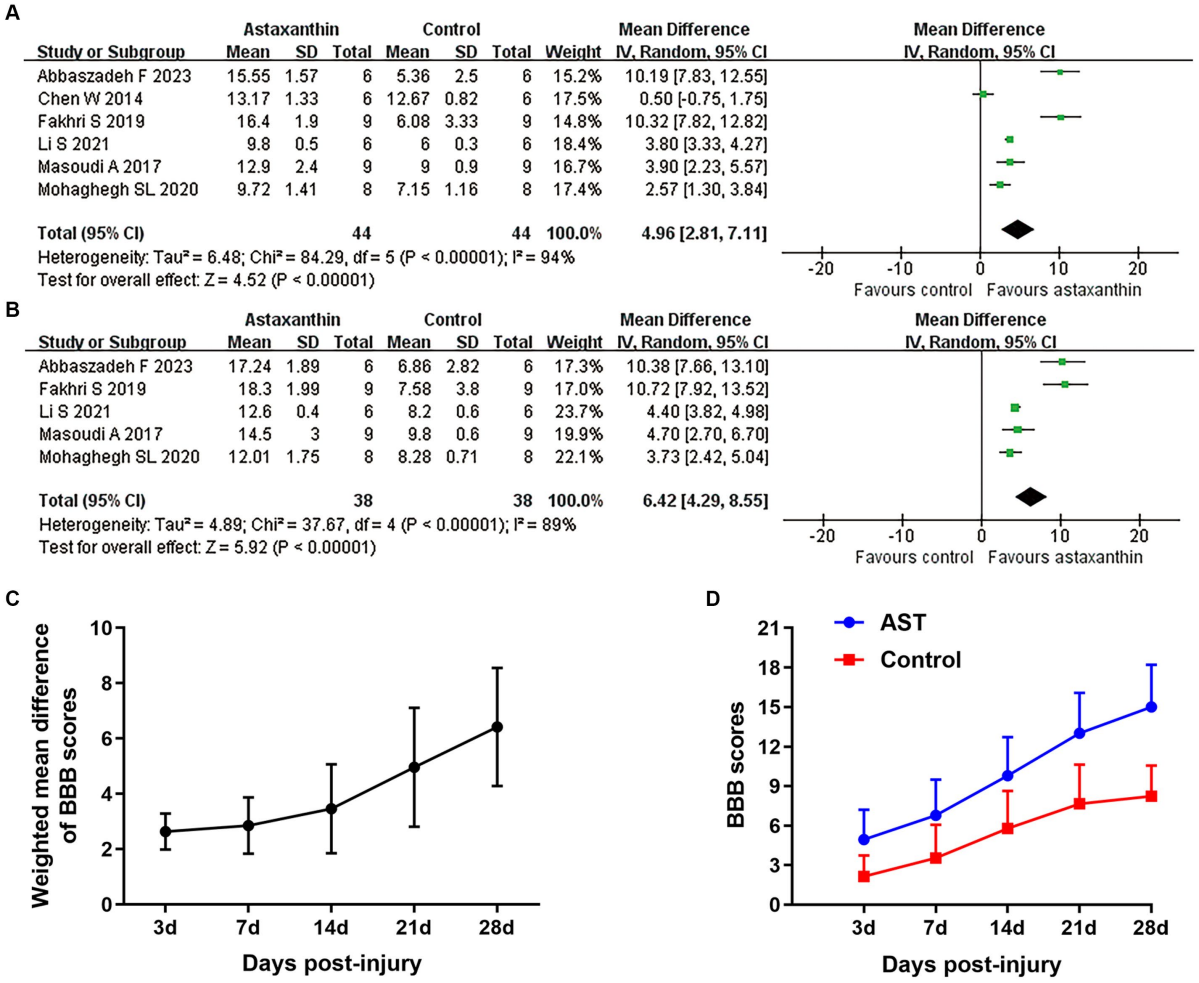
Overall analyses of the effects of AST on BBB scores. Meta-analysis of BBB scale at days 21 **(A)** and 28 **(B)** after SCI. **(C)** The WMDs of BBB score between AST and control groups from days 3 to days 28 after SCI. **(D)** The BBB scores in each group over time. The detailed data at each time point are shown in Table 4.

A total of seven studies used the BBB scale to indicate the neurological function recovery in rat models of SCI (Chen, 2014; Masoudi et al., 2017; Fakhri et al., 2019; Ren et al., 2019; Mohaghegh et al., 2020; Li et al., 2021). The meta-analyses and dynamic BBB scores in both groups suggested that BBB scores were increased in rats treated with AST compared with controls, and WMDs between those two groups exhibited a gradual upward trend from days 7 (six studies, *n* = 88, WMD = 2.85, 95% CI = 1.83 to 3.87, *p* < 0.00001) to days 28 (five studies, *n* = 76, WMD = 6.42, 95% CI = 4.29 to 8.55, *p* < 0.00001) after treatment (Figures 2A–D and Table 4).

**Table 4:** Summary of overall analyses of the effects of AST.

Outcome	No. of studies	No. of animals	Weighted mean difference	Heterogeneity		
			95% CI	*P* value	*I* ^2^	*P* value
1 BBB scores	7	100				
1.1 BBB scale at day 3	4	58	2.64 [1.99, 3.29]	< 0.00001	31	0.22
1.2 BBB scale at day 7	6	88	2.85 [1.83, 3.87]	< 0.00001	72	0.003
1.3 BBB scale at day 14	6	88	3.46 [1.85, 5.07]	< 0.0001	90	< 0.00001
1.4 BBB scale at day 21	6	88	4.96 [2.81, 7.11]	< 0.00001	94	< 0.00001
1.5 BBB scale at day 28	5	76	6.42 [4.29, 8.55]	< 0.00001	89	< 0.00001
2 Spared white matter	4	28	0.08 [0.02, 0.14]	0.007	78	0.003
3 Number of anterior horn motor neurons	4	28	7.96 [4.35, 11.57]	< 0.0001	61	0.05
4 SOD	4	40	5.29 [2.12, 8.46]	0.001	74	0.010
5 MDA	4	40	−4.20 [−6.88, −1.51]	0.002	75	0.008

**Figure 3:**
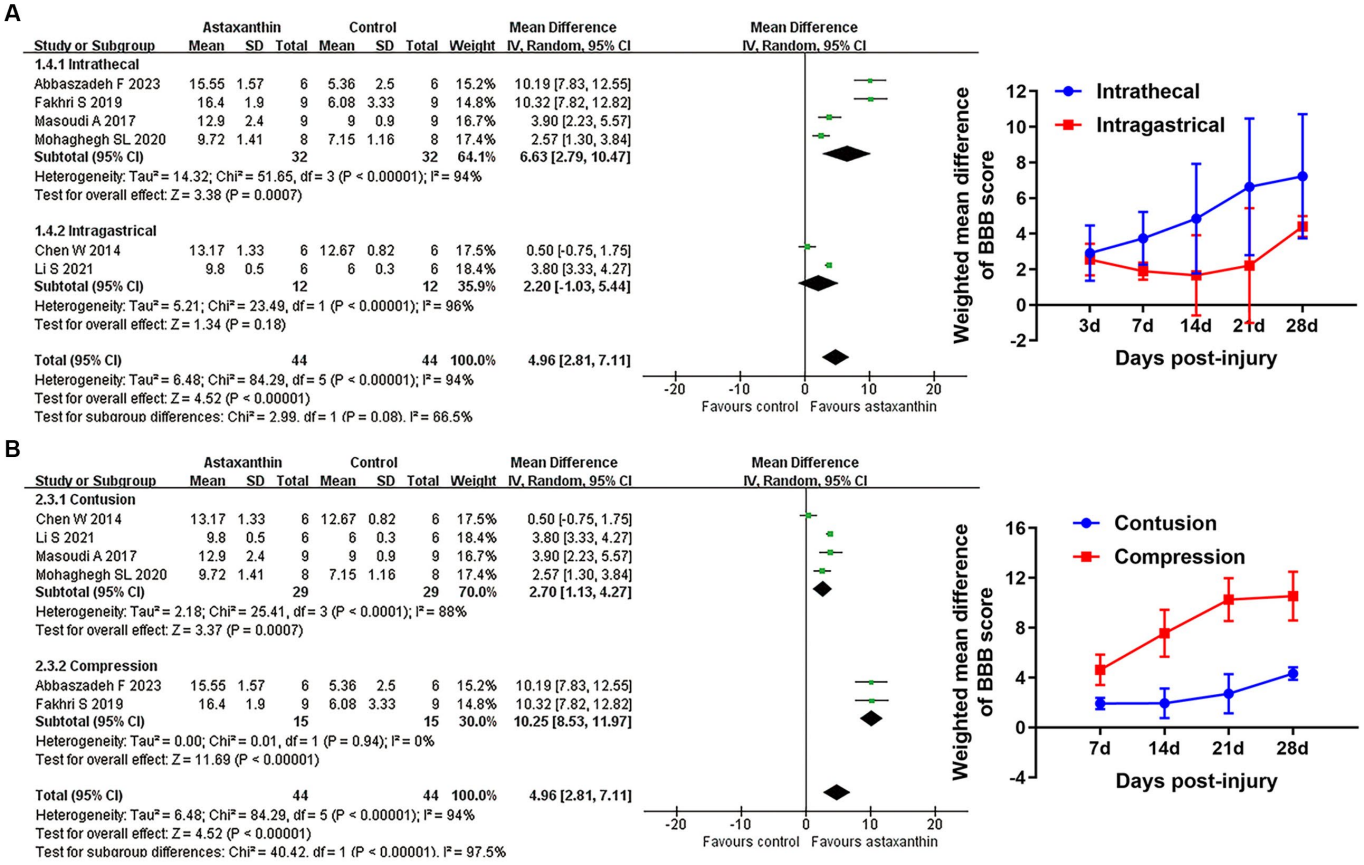
Subgroup analyses of the effects of AST on BBB scores. **(A,B)** Subgroup analysis concerning administration route and injury type at days 21 after SCI. The detailed data at each time point are shown in Table 5.

Subgroup analyses indicated that the improvement of BBB scores was associated with injury model, but did not differ between distinct administration routes (Figures 3A,B and Supplementary Table S1). Recent studies suggest that locomotor function and pathological changes may differ between rats based on gender, injury level, and timing of administration, and these factors were consistent in our included studies (Wilcox et al., 2017). Despite the different AST dosage and number of administrations among the studies, changes of those variables were highly attributed to the administration route (Chen, 2014; Ren et al., 2019; Li et al., 2021). Thus, subgroup analyses concerning those variables was not conducted in our study.

### Effects of AST on tissue sparing

**Figure 4:**
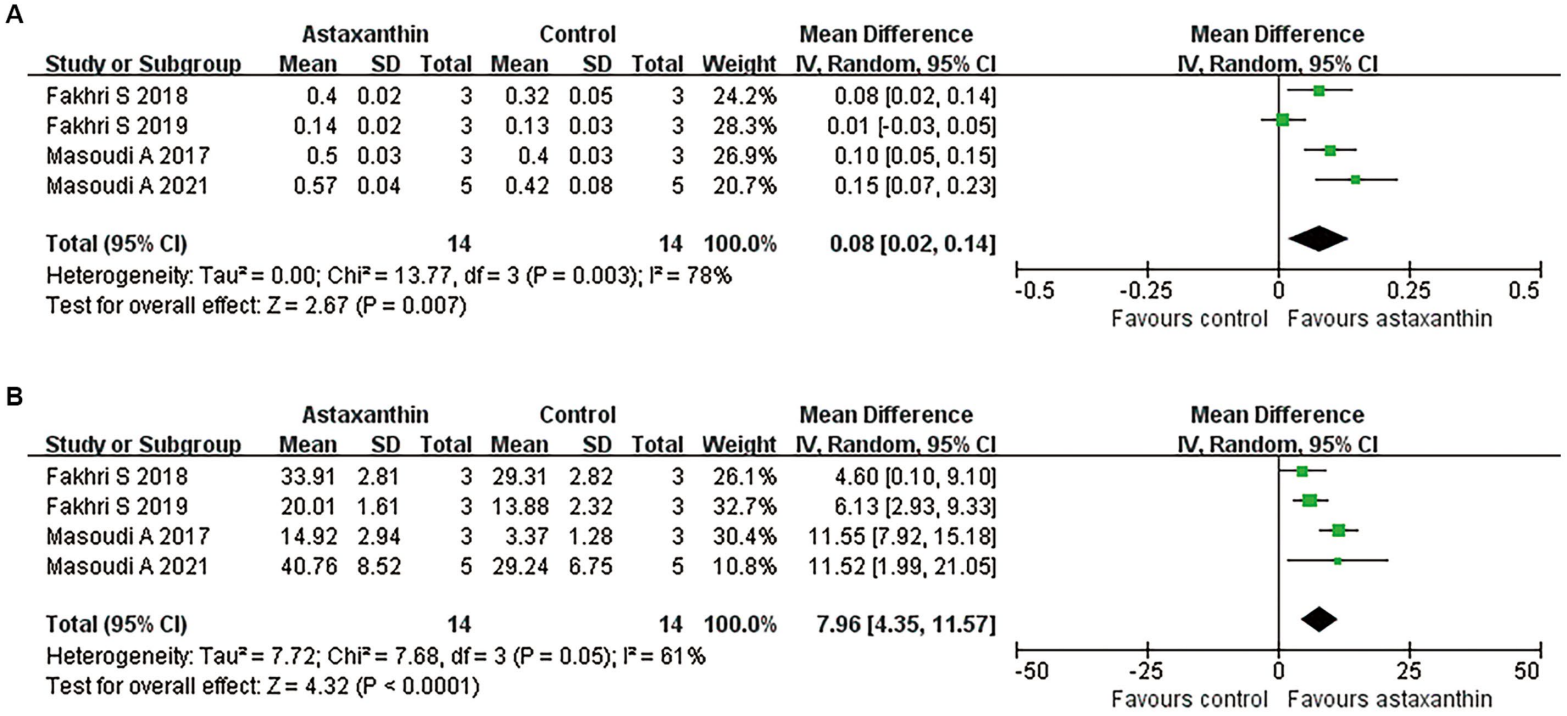
Meta-analysis of the effects of AST on tissue damage in lesion areas. Meta-analysis regarding spared white matter area **(A)** and the number of surviving motor neurons **(B)** in lesion areas at days 7 after SCI.

By using the luxol fast blue method, five studies described the spared white matter area in the form of residual tissue/cross-sectional area ratio (Masoudi et al., 2017; Fakhri et al., 2018, 2019; Mohaghegh et al., 2020; Masoudi et al., 2021). The pooled results of spared white matter area showed an increased percentage of spared white matter in AST-treated rats versus controls (four studies, *n* = 28, WMD = 0.08, 95% CI = 0.02 to 0.14, *p* = 0.007) at days 7 after SCI (Figure 4A and Table 4).

Meanwhile, five studies reported the number of motor neurons in the ventral horn area of the spinal cord by counting the Nissl positive cells (Masoudi et al., 2017; Fakhri et al., 2018, 2019; Mohaghegh et al., 2020; Masoudi et al., 2021). Meta-analysis regarding this outcome revealed an improved neuron loss in lesion areas in rats with AST intervention (Figure 4B and Table 4; four studies, *n* = 28, WMD = 7.96, 95% CI = 4.35 to 11.57, *p* < 0.0001).

### Antioxidant roles of AST

**Figure 5:**
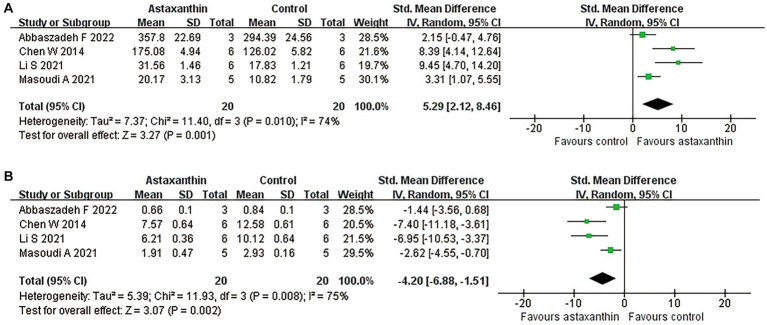
Meta-analysis of the antioxidant effects of AST. Meta-analysis regarding SOD **(A)** and MDA **(B)** in rats at day 1 following SCI.

Four studies reported the antioxidant role of AST through detecting the MDA or SOD levels in SCI rats (Chen, 2014; Li et al., 2021; Masoudi et al., 2021; Abbaszadeh et al., 2022). The units for each outcome were slightly different in those studies. Meta-analysis suggested an enhancement of SOD expression (four studies, *n* = 40, SMD = 5.29, 95% CI = 2.12 to 8.46, *p* = 0.001) and reduced MDA levels (four studies, *n* = 40, SMD = −4.20, 95% CI = −6.88 to-1.51, *p* = 0.002) in rats with AST treatment compared with control ones (Figure 5 and Table 4).

### TSA results

**Figure 6:**
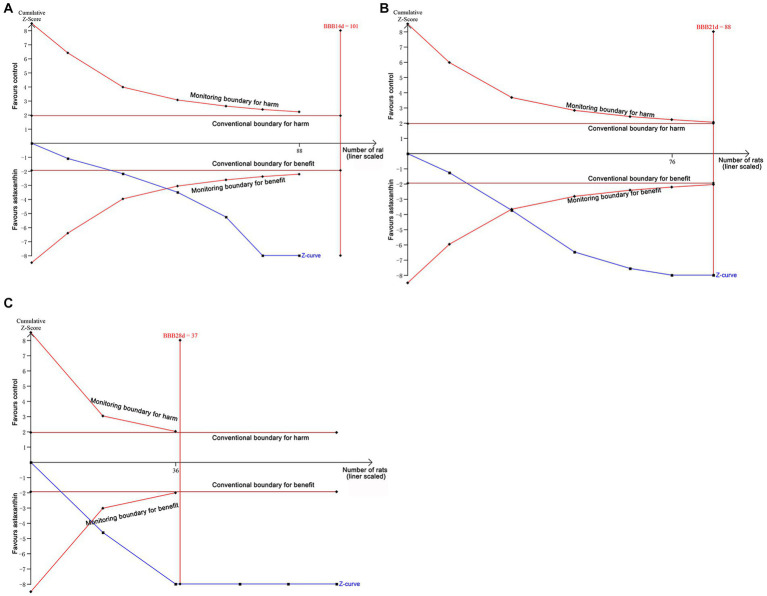
Results of TSA. TSA based on data of BBB scores at days 14 **(A)**, 21 **(B)**, and 28 **(C)** after SCI.

Data of BBB scores from days 14 to 28 after SCI were included in TSA to present the dynamic change of results. At days 14, the cumulative Z-curve crossed the monitoring boundaries, indicating although the cumulative sample size did not reach the expected value, a firm conclusion that AST could promote the recovery of locomotor function was obtained in advance (Figure. 6A). Interestingly, at the time points of days 21 and days 28, the cumulative Z-curve crossed both the conventional and monitoring boundaries, and the cumulative sample size reached the expected value (Figures 6B,C); thus, the present data firmly proved that AST can provide a beneficial role for the neurofunctional recovery of rats.

### Sensitivity analysis

Sensitivity analyses were performed by excluding either studies without outcome assessor blinding, small-sample-sized studies, or all single studies. A moderate or high heterogeneity was remained after application of all exclusion strategies. Furthermore, the improvement of BBB scores with AST remained largely unchanged (Supplementary Table S2), indicating a robust result of the analyses of BBB scores.

### Therapeutic mechanisms

The proposed mechanisms behind AST in the included studies are presented in Table 5. In general, AST exert roles of regulating oxidative stress, suppressing neuroinflammation, relieving neuron loss, and modulating autophagy in treating SCI. The N-methyl-D-aspartate receptors (NMDAR), protein kinase B (AKT), mitogen-activated protein kinase (MAPK), cleaved caspase-3, and light chain 3 (LC3) signaling may be involved in those effects of AST.

**Table 5:** The proposed mechanism of the protective effect of AST for SCI.

Study	Treatment dose; duration	Proposed mechanism	Effects
Abbaszadeh et al. (2023)	0.005 mg/kg i.t.; 30 min after injury, once	Inflammation	1. Decreased MMP-9, GFAP, and AQP4 2. Decreased HMGB1, TLR4, and NF-κB
Abbaszadeh et al. (2022)	0.005 mg/kg i.t.; 30 min after injury, once	Oxidative stress, autophagy, and apoptosis	1. Increased SOD, GSH-Px, and TAC, and decreased MDA 2. Increased Beclin1 and LC3B and decreased p62 3. Decreased Bax/Bcl2 ratio
Masoudi et al. (2021)	0.005 mg/kg i.t.; 30 min after injury, once	Oxidative stress and inflammation	1. Increased SOD, GSH-Px, and CAT, and decreased MDA 2. Decreased Cox-2, IL-1β, IL-6, and TNF-α
Li et al. (2021)	75 mg/kg i.g.; immediately, twice daily until sacrifice	Oxidative stress and apoptosis	1. Increased SOD and decreased MDA 2. Increased TUNEL positive cells
Mohaghegh et al. (2020)	0.005 mg/kg i.t.; 30 min after injury, once	Apoptosis	Increased Nissl positive cells
Fakhri et al. (2019)	0.005 mg/kg i.t.; 30 min after injury, once	Apoptosis	Modulating p-AKT/AKT and p-ERK/ERK ratio, and increased Nissl positive cells
Ren et al. (2019)	75 mg/kg i.g.; immediately, twice daily until sacrifice	Oxidative stress and inflammation	1. Decreased MPO 2. Decreased IL-1β, IL-6, and TNF-α
Fakhri et al. (2018)	0.005 mg/kg i.t.; 30 min after injury, once	Inflammation and apoptosis	1. Decreased NR2B, p-p38 MAPK, and TNF-α 2. Increased Nissl positive cells
Masoudi et al. (2017)	0.005 mg/kg i.t.; 30 min after injury, once	Apoptosis	Decreased Bax and cleaved caspase-3, and increased Bcl-2
Chen (2014)	35 mg/kg i.g.; 5 min after injury, twice daily until sacrifice	Oxidative stress and astrogliosis	1. Increased SOD and decreased MDA 2. Decreased GFAP

AQP4, Aquapurin-4; Bax, Bcl-2-associated x protein; Bcl-2, B cell lymphoma-2; CAT, catalase; Cox-2, cyclooxygenase-2; GFAP, glial fibrillary acidic protein; GSH-Px, glutathione peroxidase; HMGB1, high mobility group box-1; MDA, malondialdehyde; MMP-9, matrix Metalloproteinase-9; MPO, myeloperoxidase; NF-κB, nuclear factor-kappa B; NR2B, n-methyl-D-aspartate receptor subunit 2B; IL-1β, interleukin-1 beta; LC3B, light chain 3B; SOD, superoxide dismutase; TAC, total antioxidant capacity; TLR4, toll-like receptor 4; TNF-α, tumor necrosis factor-α; TUNEL, terminal dexynucleotidyl transferase (TdT) mediated dUTP nick end labeling; p-AKT, phosphorylated protein kinase B; p-ERK, phosphorylated extracellular signal regulated kinase; p-p38 MAPK, phosphorylated p38 mitogen-activated protein kinase.

## Discussion

An understanding of efficacy, safety, and pharmacological mechanism is critical for the translation of novel treatments to the clinic (Sandborn et al., 2020). In light of the reported beneficial roles of AST for SCI, we performed a comprehensive review of the current studies to evaluate those parameters of AST and its potential for clinical translation.

### Summary of evidence

This review extracted data from 10 studies that compared AST with placebo controls. Although most of these studies were of moderate-to-high methodological quality, the quality of evidence in these studies was not high.

The meta-analyses showed an increase in BBB scores in rats with AST treatment, and the WMDs between AST and control groups displayed a gradual upward trend over time. There were no statistical differences in locomotor recovery with respect to administration route; however, there were differences related to the injury type. Importantly, TSA firmly proved that AST can promote locomotor recovery of rats with SCI.

Compared with controls, rats in the AST groups exhibited an increased percentage of spared white matter in lesion areas. The meta-analysis concerning the motor neuron numbers confirmed the role of AST on tissue sparing. Moreover, meta-analyses revealed that AST could enhance SOD levels and downregulated the MDA expression in rats subjected to SCI, indicating an antioxidant effects of AST for SCI.

### Statistical heterogeneity and explanatory variables

As anticipated, we observed a substantial between-studies heterogeneity on treatment effects, which may reduce the credibility of the results from the analyses to a certain extent. The differences in animal characteristics, injury types, administration details, statistical methods, and methodological quality between studies may serve as potential contributors to the observed heterogeneity. Our subgroup analyses with respect to distinct injury types effectively reduced the heterogeneity in both subgroups, and the Cochrane’s I^2^ in the subgroup of compression were stably maintained at 0% from days 7 to 28 after SCI. However, it is important to keep in mind that subgroup analysis will only be hypothesis-generating and serves as an initial project to shed light on this issue. Sensitive analysis is also beneficial for exploring the sources of heterogeneity, while our sensitivity analyses using the above exclusion strategies did not successfully reduce the treatment effect heterogeneity. Additionally, three included studies administrated AST by intragastrical route; the remaining studies used intrathecal injection. Following oral absorption, AST has been shown to gradually convert into diversified metabolites (Kistler et al., 2002). The different metabolites of AST between intragastrical and intrathecal administration routes may produce various pharmacologic roles, leading to the heterogeneity in treatment effects. Collectively, a number of variables may be involved in the high heterogeneity among studies; distinct injury types and varied administration routes appear to be important factors modifying the heterogeneity in our meta-analyses. Due to the high heterogeneity among studies, the results in this review need to be interpreted cautiously.

### Effects of AST on distinct injury models

The injury mechanism is an important factor that may impact the efficacy of the treatment (Abdullahi et al., 2017). Our subgroup analyses indicated a superior locomotor recovery in compression SCI models, implying that AST may be more suitable for SCI caused by compression injury. The distinct pathophysiological mechanisms behind different injury types may account for the changed efficacy of AST treatment. Although compression and contusion SCI show a variety of similarities in the pathological changes, the compression model has its own features due to the prolonged compression of the spinal cord (Abdullahi et al., 2017). The prolonged compression can cause a decrease in blood flow, and a rapid drop in glucose level and oxygen supply in the compression site, leading to gradual ischemia damage of the spinal cord (Ueta et al., 1992; Okon et al., 2013). Subsequently, reperfusion events result in an enlarged area of damage. The ischemia and reperfusion courses rupture the oxygen or glucose supply at the injury site, and serve as typical events causing excessive oxidative stress (Ling et al., 2021). Thus, oxidative stress may act as a more prominent element in compression SCIs compared with contusion ones. As described above, AST stands out among its chemical family for its excellent antioxidant activities (Alugoju et al., 2022). Thus, we hypothesized that the more prominent oxidative stress in compression injuries may result in the superior applicability of AST on this injury type. However, this hypothesis needs further studies for verification.

### Bioavailability and pharmacokinetics

#### Bioavailability

Despite the well-documented biological activities, the bioavailability of AST is relatively low due to its hydrophobicity and poor dissolution in the gastrointestinal tract (Fratter et al., 2019). Several studies have been attempted to improve the bioavailability of AST. Among them, multiple animal studies described an enhanced bioavailability and antioxidant role of AST when administrated alongside vegetable or fish oil (Otton et al., 2012; Xu et al., 2017). The enhanced oral bioavailability of AST was confirmed in human trials by incorporating AST in lipid-based formulations (Mercke et al., 2003). Delivery of AST through liposomes or nanocarriers has also been shown to promote the absorption of AST in recent years (Zhang et al., 2021; Srihera et al., 2022), while those delivery strategies also warrant further investigation in the field of SCI. Additionally, intrathecal injection of AST may offer an alternative option to enhance the bioavailability for central nervous system (CNS) diseases (Abbaszadeh et al., 2022). In our meta-analyses, although there were no significant differences in BBB scores between intrathecal injection or intragastrical administration at most time points, the intrathecal injection of AST appeared to display a more obvious tendency in BBB scores improvement. Given the above rationale, administration with oils and a lipid-based delivery system, or through liposomes and nanocarriers, may provide possible options for enhancing the bioavailability and biological effects of AST. Furthermore, intrathecal injection of AST seems to be an optional strategy for SCI treatment.

#### Pharmacokinetics

Distribution in the CNS *via* crossing the blood–brain barrier is powerful evidence to imply a drug’s effects on the CNS. AST is one of the few carotenoids that can cross the blood–brain barrier. Pharmacokinetic studies revealed that oral administration of AST can lead to a drug accumulation in rat brain tissues rapidly, and achieve a higher drug concentration in the brain compared with those in plasma, heart, or lungs at 8 and 24 h after administration (Choi et al., 2011). Meanwhile, rats with repeated consumption of AST exhibited a higher concentration in the brain compared with ones administered a single dose (Manabe et al., 2018). In humans, the absorption and metabolism processes are closely related to the lifestyle and health conditions of subjects (Mercke et al., 2003; Bahbah et al., 2021). For example, smoking can enhance the metabolism and elimination of AST, thereby significantly reducing the half-life of AST, while administration of AST after a meal downregulates the elimination rate and enhances the max blood concentration in humans (Okada et al., 2014). Despite an absence of AST pharmacokinetic parameters in SCI, the above findings indicated that repeated administration, controlling lifestyle, and administration modifications based on pharmacokinetic monitoring may need to be considered in the research regarding AST for SCI.

### Safety

AST is approved by the Food and Drug Administration and European Food Safety Authority as a food supplement. The adverse effects of AST are rare, involving the alteration of skin pigmentation, increased frequency in bowel movements, and coloration change of feces (Brendler and Williamson, 2019). Although excessive consumption of AST is associated with potential side effects, a large range between the median effective dose and the median lethal dose has been indicated in animal experiments. For instance, oral administration of AST at doses of 5–75 mg/kg all exerted neuroprotective roles in rats without death events caused by AST (Zhang et al., 2014; Liu H. et al., 2020); even at consumption levels of 20 g/kg AST, the majority of the treated animals exhibited a normal condition in clinical parameters (Niu et al., 2020). Meanwhile, only minor clinical differences in blood indexes and slight hepatotoxicity were observed in rats continuously treated by 1,000 mg/kg AST for two years (Edwards et al., 2016). In human trials, antioxidant and anti-inflammatory benefits were achieved with as little as 2 mg/day, and no serious adverse events were observed with a high dose of 45 mg/day for four weeks (Kajita et al., 2010; Park et al., 2010). A single dose of 100 mg AST was once applied in humans to investigate the pharmacokinetics of AST, but no serious events were presented in this study (Østerlie et al., 2000). Furthermore, the European Food Safety Authority concluded that a daily intake of 8 mg AST from food supplements is safe for adults even in combination with the high exposure estimate to AST from the background diet (Turck et al., 2020). However, the safety of AST for patients with SCI remains to be confirmed due to a lack of related human clinical trials.

### Potential therapeutic mechanisms

**Figure 7:**
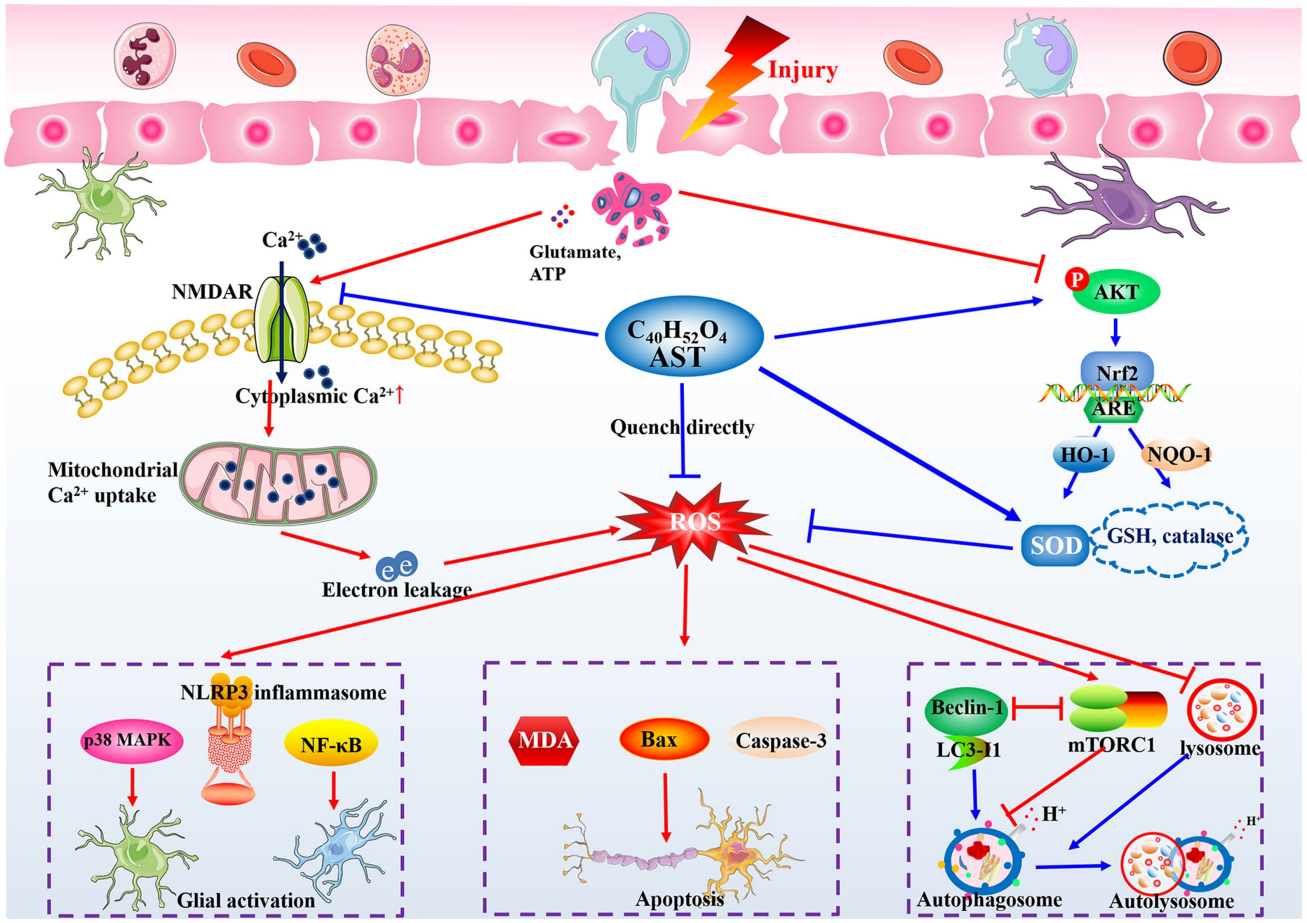
Potential action mechanisms of AST in treating SCI. The oxidative stress and large generation of ROS are immediately triggered following the insult to the spinal cord, and can lead to the neuroinflammatory response, neuronal apoptosis, and organelle dysfunction. AST shows satisfactory roles on controlling oxidative stress generation in CNS diseases through its unique construction, and modulating NMDAR and Nrf2 signaling pathways. Partly relying on its antioxidant bioactivity, AST then inhibits MAPK, NLRP3 inflammasome, or NF-κB signaling to alleviate the local inflammation, modulate the Bax and caspase family proteins to promote neuron survival, and stabilize the autophagy flux by regulating beclin-1 and mTOR complex 1, finally exerting neuroprotective roles in treating SCI rats.

Based on the proposed mechanisms in the included studies and related evidence, the potential mechanisms of AST are summarized as follows (Figure 7).

#### Eliminating oxidative stress

Rapidly initiated and persistent oxidative stress plays a crucial role in the pathological process of SCI (Feng et al., 2021; Zrzavy et al., 2021). SOD is the first line of defense against oxygen free radicals. It can catalyze the dismutation of superoxide to form hydrogen peroxide and oxygen, limiting the chemical toxicity of ROS (Wang et al., 2018). MDA mainly originates from lipid peroxidation reaction mediated by ROS, then it participates in secondary deleterious reactions and enhances ROS generation in return (Wang et al., 2019). Our pooled results indicated the beneficial role of AST on SOD and MDA levels at day 1 following injury, implying AST favorably regulates the acute oxidative stress in SCI. It is argued that the unique structure endows AST with a powerful antioxidative property. AST contains conjugated double bonds and hydroxyl and keto groups, whereby it quenches singlet oxygen and scavenges ROS directly by donating electrons or attracting unpaired electrons (Brotosudarmo et al., 2020). According to the data in the included studies, several signaling pathways serve as intermediary links for AST to exert its antioxidant roles. The Ca^2+^ influx caused by NMDAR activation can destroy the homeostasis of the mitochondrial respiratory chain, leading to electron leakage and the large generation of ROS in CNS injuries. Interestingly, AST stably suppressed NMDAR activation from days 7 to days 28 post-injury in SCI rats (Fakhri et al., 2018). Meanwhile, compared with the vehicle ones, SCI rats treated with AST exhibited a markedly increased phosphorylation level of AKT (Fakhri et al., 2019), which is critical for increasing SOD and lowering MDA through nuclear factor-erythroid 2-related factor 2 to defend against oxidative stress (Zhao et al., 2017).

#### Counteracting neuroinflammation

Following trauma, the microglia, astrocytes, and peripheral immune cells can be rapidly activated to induce an inflammatory cell cascade (Salvador and Kipnis, 2022). Oxidative stress is demonstrated to be an important signal to trigger inflammatory cascade responses (Liu Z. et al., 2020). Correspondingly, multiple of the included studies revealed AST can mitigate the inflammatory responses in SCI by lowering the expression of cyclooxygenase-2 and pro-inflammatory cytokines (Masoudi et al., 2021). Glial cell activation was also ameliorated by AST to impede the inflammatory response in SCI models. The activation of MAPK, nuclear transcription factor-κB (NF-κB), or the nod-like receptor family pyrin domain-containing 3 (NLRP3) inflammasome is an important downstream signal of ROS, mediating the persistent inflammatory responses during SCI (Liu Z. et al., 2020). Fakhri et al. (2018) demonstrated AST exhibited a persistent role in lowing p38 MAPK phosphorylation in the lesion areas of SCI (Fakhri et al., 2018). The downregulated roles of AST on NF-κB and NLRP3 inflammasome signaling were also confirmed in related CNS diseases (Zhuang et al., 2021). Thus, inhibition of oxidative stress and downstream signals such as MAPK and NLRP3 inflammasome may account for the role of AST in counteracting neuroinflammation.

#### Alleviating neuron loss

Excessive oxidative stress following injury can markedly perturb the function of organelles and physiological metabolism in cells, resulting in an activation of neurotoxic signaling cascades (Zrzavy et al., 2021; Ji et al., 2022). AST was reported to improve the survival of motor neurons and decrease the expression of apoptosis-related markers in the injury sites of spinal cords (Mohaghegh et al., 2020; Li et al., 2021). B cell lymphoma-2 (Bcl-2), Bcl-2-associated x protein (Bax), and caspases are involved in the neural cell death caused by oxidative stress (Liu et al., 2022), while AST can notably inhibit the activation of Bax and caspase-3 and increase the level of Bcl-2 in injury sites of SCI (Masoudi et al., 2017; Abbaszadeh et al., 2022). As mentioned above, the NMDAR activation induced by extracellular glutamate contributes to the large generation of ROS, and the AKT pathway can enhance ROS scavenging by increasing SOD and catalase levels. Interestingly, AST notably suppresses NMDAR activation in SCI rats (Fakhri et al., 2018), and activates AKT signaling at injury sites (Fakhri et al., 2019). Then, a potential molecular chain constructed by NMDAR (or AKT), ROS, Bax, and caspases signals may be involved in the mechanisms underlying the neuroprotective effects of AST in treating SCI.

#### Regulating the autophagy flux

The stimulation of autophagy flux is highly associated with microtubule stabilization, the removal of damaged organelles, and neuron survival in CNS diseases (Liao et al., 2021). Beclin1, LC3, and autophagy-related proteins are positive regulators for autolysosome formation, while excessive ROS generation from oxidative stress can impair the autophagy flux through activating mTOR complex 1 or inducing lysosomal dysfunction (Zhou et al., 2020; Liao et al., 2021). A number of studies have revealed an elevation of autophagy flux following AST treatment in animals with SCI or related CNS diseases (Abbaszadeh et al., 2022; Fu et al., 2022). Compared with the controls, rats that underwent AST treatment manifested increased levels of Beclin1 and LC3 and a decreased expression of p62 (Abbaszadeh et al., 2022). Additionally, AST was shown to restore the normal function of lysosomes and notably inhibited the activation of mTOR complex1 (Fu et al., 2022; Xiang et al., 2022), while this mechanism remains to be confirmed in SCI models.

Collectively, given the critical role of oxidative stress in the secondary injury cascade of SCI, we speculated eliminating oxidative stress may serve as the core mechanism of AST for exerting a neuroprotective effect. Partly relying on the antioxidant activity, AST then exhibits roles of regulating neuroinflammation, cell loss, and autophagy *via* multiple molecular pathways in treating SCI (Figure 7). However, due to the insufficiency of the current evidence, more studies are needed for further elucidation of the details regarding the neuroprotective mechanism of AST.

### Strengths and limitations

To our knowledge, this is the first meta-analysis quantitatively analyzing the efficacy of AST in SCI. This review primarily focused on the dynamic changes of BBB scores following AST intervention, and dealt with the tissue protective and antioxidant effects of AST in SCI. TSA confirmed the results from our meta-analyses and firmly supported the beneficial effects of AST. Given the significance of efficacy, safety, pharmacokinetics, and pharmacological mechanisms for effective clinical translation, we employed a combination of a systematic and traditional review to comprehensively analyze all of these parameters. The findings in this review suggest that AST is a promising natural neuroprotective agent for SCI treatment.

There are several limitations to our study. First, the included studies were still limited although an extensive search strategy was applied, which may challenge the results and meaning of our meta-analysis. However, analyzing the dynamic changes of BBB scores and using the multimodal assessments (assessor evaluation, biochemistry indicators, and histopathological examinations) were beneficial for indicating the actual effects of AST. Additionally, the TSA analyses further confirmed the results from our meta-analyses. Second, substantial heterogeneity between studies was presented in our review. The heterogeneity appears to be linked to the differences in injury type, administration route, and other above factors, and may lead to improper understanding of the efficacy of AST. However, the random effect model widely used in our analyses reduced the risk of producing incorrect results to a certain degree. Meanwhile, the robust results in sensitivity analyses and consistent result tendencies among the studies enhanced the strength of evidence regarding AST efficacy. Third, systematic subgroup analyses are important for investigating the efficacy of AST in distinct scenarios. Due to the variable distribution in studies and the limited number of included studies, subgroup analyses with respect to rat gender, injury level, timing, or dose of administration were not conducted. Fourth, the SYRCLE’s RoB tool showed a potential risk of bias within the included studies. It is a common problem for animal studies, but may have produced unreliable results in the included studies and our meta-analyses.

## Conclusion

In conclusion, our meta-analyses agreed that AST can ameliorate neurological function deficits and attenuate tissue damage in treating SCI. The TSA firmly proved the beneficial effects of AST on the locomotor recovery of rats following SCI. Meanwhile, AST exhibits favorable safety in numerous animal and human studies, with a rational neuroprotective mechanism. Therefore, we suggest that AST is a promising candidate for future clinical trials of SCI, which may result in a novel clinical therapy for this disease. Nonetheless, to move toward clinical trials, extensive pre-clinical studies are needed to understand the in-depth mechanisms of AST on SCI. Meanwhile, the results of this review should be interpreted cautiously due to the limitations in the design and methodological quality of the included studies.

## Data availability statement

The original contributions presented in the study are included in the article, further inquiries can be directed to the corresponding author.

## Author contributions

L-yZ: Data curation, Formal analysis, Visualization, Writing – original draft. Z-mW: Data curation, Formal analysis, Visualization, Writing – original draft. X-qC: Data curation, Methodology, Writing – original draft. B-bY: Data curation, Validation, Writing – original draft. M-xP: Methodology, Validation, Writing – original draft. LF: Data curation, Methodology, Writing – original draft. JL: Validation, Visualization, Writing – original draft. X-jC: Methodology, Validation, Writing – original draft. MY: Supervision, Writing – review & editing, Conceptualization. XL: Conceptualization, Supervision, Writing – review & editing.

## Footnotes

^1^ http://getdata-graph-digitizer.com

## References

[ref1] Abbaszadeh F. Jorjani M. Joghataei M. T. Mehrabi S. (2022). Astaxanthin modulates autophagy, apoptosis, and neuronal oxidative stress in a rat model of compression spinal cord injury. Neurochem. Res. 47, 2043–2051. doi: 10.1007/s11064-022-03593-1, PMID: 35435619

[ref2] Abbaszadeh F. Jorjani M. Joghataei M. Raminfard S. Mehrabi S. (2023). Astaxanthin ameliorates spinal cord edema and astrocyte activation via suppression of HMGB1/TLR4/NF-κB signaling pathway in a rat model of spinal cord injury. Naunyn Schmiedeberg's Arch. Pharmacol. doi: 10.1007/s00210-023-02512-7, PMID: 37145127

[ref3] Abdol W. N. Meor M. A. M. Fakurazi S. Alias E. Hassan H. (2022). Nanocarrier system: state-of-the-art in oral delivery of astaxanthin. Antioxidants (Basel). 11:1676. doi: 10.3390/antiox11091676, PMID: 36139750 PMC9495775

[ref4] Abdullahi D. Annuar A. A. Mohamad M. Aziz I. Sanusi J. (2017). Experimental spinal cord trauma: a review of mechanically induced spinal cord injury in rat models. Rev. Neurosci. 28, 15–20. doi: 10.1515/revneuro-2016-0050, PMID: 27845888

[ref5] Alugoju P. Krishna S. V. Anthikapalli N. Tencomnao T. (2022). Health benefits of astaxanthin against age-related diseases of multiple organs: a comprehensive review. Crit. Rev. Food Sci. Nutr. 1-66, 1–66. doi: 10.1080/10408398.2022.2084600, PMID: 35708049

[ref6] Anjum A. Yazid M. D. I. Fauzi Daud M. Idris J. Ng A. M. H. Selvi Naicker A. . (2020). Spinal cord injury: pathophysiology, multimolecular interactions, and underlying recovery mechanisms. Int. J. Mol. Sci. 21:7533. doi: 10.3390/ijms21207533, PMID: 33066029 PMC7589539

[ref7] Bahbah E. I. Ghozy S. Attia M. S. Negida A. Emran T. B. Mitra S. . (2021). Molecular mechanisms of astaxanthin as a potential neurotherapeutic agent. Mar. Drugs 19:201. doi: 10.3390/md19040201, PMID: 33916730 PMC8065559

[ref8] Basso D. M. Beattie M. S. Bresnahan J. C. (1995). A sensitive and reliable locomotor rating scale for open field testing in rats. J. Neurotrauma 12, 1–21. doi: 10.1089/neu.1995.12.1, PMID: 7783230

[ref9] Brendler T. Williamson E. M. (2019). Astaxanthin: how much is too much? safety review. Phytother. Res. 33, 3090–3111. doi: 10.1002/ptr.6514, PMID: 31788888

[ref10] Brotosudarmo T. Limantara L. Setiyono E. Heriyanto (2020). Structures of astaxanthin and their consequences for therapeutic application. Int. J. Food Sci. 2020:2156582. doi: 10.1155/2020/2156582, PMID: 32775406 PMC7391096

[ref11] Chao C. Yeh H. Tsai Y. Yuan T. Liao M. Huang J. . (2020). Astaxanthin counteracts vascular calcification in vitro through an early up-regulation of SOD2 based on a transcriptomic approach. Int. J. Mol. Sci. 21:8530. doi: 10.3390/ijms21228530, PMID: 33198315 PMC7698184

[ref12] Charalambous M. Brodbelt D. Volk H. A. (2014). Treatment in canine epilepsy--a systematic review. BMC Vet. Res. 10:257. doi: 10.1186/s12917-014-0257-9, PMID: 25338624 PMC4209066

[ref13] Charalambous M. Shivapour S. K. Brodbelt D. C. Volk H. A. (2016). Antiepileptic drugs’ tolerability and safety-a systematic review and meta-analysis of adverse effects in dogs. BMC Vet. Res. 12:79. doi: 10.1186/s12917-016-0703-y, PMID: 27206489 PMC4875685

[ref14] Chen W. (2014). Research the protective function and related mechanism of astaxanthin in early spinal cord injury in SD rats. Hengyang: University of South China.

[ref15] Choi H. D. Kang H. E. Yang S. H. Lee M. G. Shin W. G. (2011). Pharmacokinetics and first-pass metabolism of astaxanthin in rats. Brit. J. Nutr. 105, 220–227. doi: 10.1017/S0007114510003454, PMID: 20819240

[ref16] Donoso A. González-Durán J. Muñoz A. A. González P. A. Agurto-Muñoz C. (2021). Therapeutic uses of natural astaxanthin: an evidence-based review focused on human clinical trials. Pharmacol. Res. 166:105479. doi: 10.1016/j.phrs.2021.105479 33549728

[ref17] Edwards J. A. Bellion P. Beilstein P. Rümbeli R. Schierle J. (2016). Review of genotoxicity and rat carcinogenicity investigations with astaxanthin. Regul. Toxicol. Pharmacol. 75, 5–19. doi: 10.1016/j.yrtph.2015.12.009, PMID: 26713891

[ref18] Fakhri S. Abbaszadeh F. Dargahi L. Pouriran R. Jorjani M. (2022). Astaxanthin ameliorates serum level and spinal expression of macrophage migration inhibitory factor following spinal cord injury. Behav. Pharmacol. 33, 505–512. doi: 10.1097/FBP.0000000000000698, PMID: 36148838

[ref19] Fakhri S. Dargahi L. Abbaszadeh F. Jorjani M. (2018). Astaxanthin attenuates neuroinflammation contributed to the neuropathic pain and motor dysfunction following compression spinal cord injury. Brain Res. Bull. 143, 217–224. doi: 10.1016/j.brainresbull.2018.09.011, PMID: 30243665

[ref20] Fakhri S. Dargahi L. Abbaszadeh F. Jorjani M. (2019). Effects of astaxanthin on sensory-motor function in a compression model of spinal cord injury: involvement of ERK and AKT signalling pathway. Eur. J. Pain 23, 750–764. doi: 10.1002/ejp.1342, PMID: 30427581

[ref21] Feng Z. Min L. Chen H. Deng W. Tan M. Liu H. . (2021). Iron overload in the motor cortex induces neuronal ferroptosis following spinal cord injury. Redox Biol. 43:101984. doi: 10.1016/j.redox.2021.101984, PMID: 33933882 PMC8105676

[ref22] Fratter A. Biagi D. Cicero A. (2019). Sublingual delivery of astaxanthin through a novel ascorbyl palmitate-based nanoemulsion: preliminary data. Mar. Drugs 17:508. doi: 10.3390/md17090508, PMID: 31470537 PMC6780925

[ref23] Fu M. Liang X. Zhang X. Yang M. Ye Q. Qi Y. . (2022). Astaxanthin delays brain aging in senescence-accelerated mouse prone 10: inducing autophagy as a potential mechanism. Nutr. Neurosci. 26, 445–455. doi: 10.1080/1028415X.2022.2055376, PMID: 35385370

[ref24] Gbd N. C. (2019). Global, regional, and national burden of neurological disorders, 1990-2016: a systematic analysis for the global burden of disease study 2016. Lancet Neurol. 18, 459–480. doi: 10.1016/S1474-4422(18)30499-X, PMID: 30879893 PMC6459001

[ref25] Higgins J. Green S. (2011). Cochrane handbook for systematic reviews of interventions version 5.1.0. The Cochrane collaboration Available at: www.handbook.cochrane.org.

[ref26] Hooijmans C. R. Rovers M. M. de Vries R. B. Leenaars M. Ritskes-Hoitinga M. Langendam M. W. (2014). SYRCLE's risk of bias tool for animal studies. BMC Med. Res. Methodol. 14:43. doi: 10.1186/1471-2288-14-43, PMID: 24667063 PMC4230647

[ref27] Ji Z. Gao G. Ma Y. Luo J. Zhang G. Yang H. . (2022). Highly bioactive iridium metal-complex alleviates spinal cord injury via ROS scavenging and inflammation reduction. Biomaterials 284:121481. doi: 10.1016/j.biomaterials.2022.121481, PMID: 35405576

[ref28] Jiang B. Sun D. Sun H. Ru X. Liu H. Ge S. . (2022). Prevalence, incidence, and external causes of traumatic spinal cord injury in China: a nationally representative cross-sectional survey. Front. Neurol. 12:784647. doi: 10.3389/fneur.2021.784647, PMID: 35126291 PMC8811043

[ref29] Kajita M. Kato T. Yoshimoto T. Masuda K. (2010). Study on the safety of high-dose administration of astaxanthin. Folia Japonica de Ophthalmologica Clinica. 3, 365–370.

[ref30] Kistler A. Liechti H. Pichard L. Wolz E. Oesterhelt G. Hayes A. . (2002). Metabolism and CYP-inducer properties of astaxanthin in man and primary human hepatocytes. Arch. Toxicol. 75, 665–675. doi: 10.1007/s00204-001-0287-5, PMID: 11876499

[ref31] Li S. Gao X. Zhang Q. Zhang X. Lin W. Ding W. (2021). Astaxanthin protects spinal cord tissues from apoptosis after spinal cord injury in rats. Ann Transl Med. 9:1796. doi: 10.21037/atm-21-6356, PMID: 35071490 PMC8756234

[ref32] Liao H. Wang Z. Ran R. Zhou K. Ma C. Zhang H. (2021). Biological functions and therapeutic potential of autophagy in spinal cord injury. Front. Cell Dev. Biol. 9:761273. doi: 10.3389/fcell.2021.761273, PMID: 34988074 PMC8721099

[ref33] Ling X. Lu J. Yang J. Qin H. Zhao X. Zhou P. . (2021). Non-coding RNAs: emerging therapeutic targets in spinal cord ischemia–reperfusion injury. Front. Neurol. 12:680210. doi: 10.3389/fneur.2021.680210, PMID: 34566835 PMC8456115

[ref34] Liu D. Liu J. Sun D. Wen J. (2004). The time course of hydroxyl radical formation following spinal cord injury: the possible role of the Iron-catalyzed Haber-Weiss reaction. J. Neurotraum. 21, 805–816. doi: 10.1089/0897715041269650, PMID: 15253806

[ref35] Liu H. Zhang X. Xiao J. Song M. Cao Y. Xiao H. . (2020). Astaxanthin attenuatesd -galactose-induced brain aging in rats by ameliorating oxidative stress, mitochondrial dysfunction, and regulating metabolic markers. Food Funct. 11, 4103–4113. doi: 10.1039/D0FO00633E 32343758

[ref36] Liu Y. Meng X. Sun L. Pei K. Chen L. Zhang S. . (2022). Protective effects of hydroxy-α-sanshool from the pericarp of Zanthoxylum bungeanum maxim. On D-galactose/AlCl3-induced Alzheimer's disease-like mice via Nrf2/HO-1 signaling pathways. Eur. J. Pharmacol. 914:174691. doi: 10.1016/j.ejphar.2021.174691 34896111

[ref37] Liu Z. Yao X. Jiang W. Li W. Zhu S. Liao C. . (2020). Advanced oxidation protein products induce microglia-mediated neuroinflammation via MAPKs-NF-κB signaling pathway and pyroptosis after secondary spinal cord injury. J. Neuroinflamm. 17:90. doi: 10.1186/s12974-020-01751-2, PMID: 32192500 PMC7082940

[ref38] Liu Z. Yao X. Sun B. Jiang W. Liao C. Dai X. . (2021). Pretreatment with kaempferol attenuates microglia-mediate neuroinflammation by inhibiting MAPKs-NF-κB signaling pathway and pyroptosis after secondary spinal cord injury. Free Radical Bio. Med. 168, 142–154. doi: 10.1016/j.freeradbiomed.2021.03.037, PMID: 33823244

[ref39] Lucchesi L. R. Agrawal S. Ahmadi A. Aichour A. N. Altirkawi K. Ariani F. . (2019). Global, regional, and national burden of traumatic brain injury and spinal cord injury, 1990–2016: a systematic analysis for the global burden of disease study 2016. Lancet Neurol. 18, 56–87. doi: 10.1016/S1474-4422(18)30415-0, PMID: 30497965 PMC6291456

[ref40] Manabe Y. Komatsu T. Seki S. Sugawara T. (2018). Dietary astaxanthin can accumulate in the brain of rats. Biosci. Biotechnol. Biochem. 82, 1433–1436. doi: 10.1080/09168451.2018.1459467, PMID: 29625535

[ref41] Masoudi A. Dargahi L. Abbaszadeh F. Pourgholami M. H. Asgari A. Manoochehri M. . (2017). Neuroprotective effects of astaxanthin in a rat model of spinal cord injury. Behav. Brain Res. 329, 104–110. doi: 10.1016/j.bbr.2017.04.026 28442361

[ref42] Masoudi A. Jorjani M. Alizadeh M. Mirzamohammadi S. Mohammadi M. (2021). Anti-inflammatory and antioxidant effects of astaxanthin following spinal cord injury in a rat animal model. Brain Res. Bull. 177, 324–331. doi: 10.1016/j.brainresbull.2021.10.014, PMID: 34688832

[ref43] Matsuda M. Kanno H. Sugaya T. Yamaya S. Yahata K. Handa K. . (2020). Low-energy extracorporeal shock wave therapy promotes BDNF expression and improves functional recovery after spinal cord injury in rats. Exp. Neurol. 328:113251. doi: 10.1016/j.expneurol.2020.113251 32087252

[ref44] Mercke O. J. Lignell A. Pettersson A. Hoglund P. (2003). Oral bioavailability of the antioxidant astaxanthin in humans is enhanced by incorporation of lipid based formulations. Eur. J. Pharm. Sci. 19, 299–304. doi: 10.1016/s0928-0987(03)00135-0, PMID: 12885395

[ref45] Mohaghegh S. L. Valian N. Pournajaf S. Abbaszadeh F. Dargahi L. Jorjani M. (2020). Combination therapy with astaxanthin and epidermal neural crest stem cells improves motor impairments and activates mitochondrial biogenesis in a rat model of spinal cord injury. Mitochondrion 52, 125–134. doi: 10.1016/j.mito.2020.03.002, PMID: 32151747

[ref46] Niu T. Zhou J. Wang F. Xuan R. Chen J. Wu W. . (2020). Safety assessment of astaxanthin from *Haematococcus pluvialis*: acute toxicity, genotoxicity, distribution and repeat-dose toxicity studies in gestation mice. Regul. Toxicol. Pharmacol. 115:104695. doi: 10.1016/j.yrtph.2020.104695, PMID: 32512118

[ref47] Okada Y. Ishikura M. Maoka T. (2014). Bioavailability of astaxanthin inHaematococcus algal extract: the effects of timing of diet and smoking habits. Biosci. Biotechnol. Biochem. 73, 1928–1932. doi: 10.1271/bbb.90078, PMID: 19734684

[ref48] Okon E. B. Streijger F. Lee J. H. Anderson L. M. Russell A. K. Kwon B. K. (2013). Intraparenchymal microdialysis after acute spinal cord injury reveals differential metabolic responses to contusive versus compressive mechanisms of injury. J. Neurotrauma 30, 1564–1576. doi: 10.1089/neu.2013.2956, PMID: 23768189

[ref49] Østerlie M. Bjerkeng B. Liaaen-Jensen S. (2000). Plasma appearance and distribution of astaxanthin E/Z and R/S isomers in plasma lipoproteins of men after single dose administration of astaxanthin. J. Nutr. Biochem. 11, 482–490. doi: 10.1016/S0955-2863(00)00104-2, PMID: 11120445

[ref50] Otton R. Marin D. P. Bolin A. P. de Cassia S. M. R. Campoio T. R. Fineto C. J. . (2012). Combined fish oil and astaxanthin supplementation modulates rat lymphocyte function. Eur. J. Nutr. 51, 707–718. doi: 10.1007/s00394-011-0250-z, PMID: 21972007

[ref51] Park J. S. Chyun J. H. Kim Y. K. Line L. L. Chew B. P. (2010). Astaxanthin decreased oxidative stress and inflammation and enhanced immune response in humans. Nutr. Metab. (Lond.) 7:18. doi: 10.1186/1743-7075-7-18, PMID: 20205737 PMC2845588

[ref52] Ren X. S. Ding W. Li Z. W. Yang X. Y. (2019). Effects of Astaxanthin on inflammation response after spinal cord injury in rats. Chinese. J. Rehabil. Med. 34:766-771, 782. doi: 10.3969/j.issn.1001-1242.2019.07.004

[ref53] Ren X. S. Ding W. Yang X. Y. (2018). Effect of astaxanthin on the apoptosis after spinal cord injury in rats. Chinese Journal of Reparative and Reconstructive Surgery. 32, 548–553. doi: 10.7507/1002-1892.201712127, PMID: 29806341 PMC8430011

[ref54] Salvador A. F. M. Kipnis J. (2022). Immune response after central nervous system injury. Semin. Immunol. 59:101629. doi: 10.1016/j.smim.2022.101629 35753867

[ref55] Sandborn W. J. Nguyen D. D. Beattie D. T. Brassil P. Krey W. Woo J. . (2020). Development of gut-selective Pan-Janus kinase inhibitor TD-1473 for ulcerative colitis: a translational medicine programme. J. Crohn's Colitis 14, 1202–1213. doi: 10.1093/ecco-jcc/jjaa049, PMID: 32161949 PMC7493219

[ref56] Scheijen E. Hendrix S. Wilson D. R. (2022). Oxidative DNA damage in the pathophysiology of spinal cord injury: seems obvious, but where is the evidence? Antioxidants (Basel). 11:1728. doi: 10.3390/antiox11091728, PMID: 36139802 PMC9495924

[ref57] Sng K. S. Li G. Zhou L. Y. Song Y. J. Chen X. Q. Wang Y. J. . (2022). Ginseng extract and ginsenosides improve neurological function and promote antioxidant effects in rats with spinal cord injury: a meta-analysis and systematic review. J. Ginseng Res. 46, 11–22. doi: 10.1016/j.jgr.2021.05.009, PMID: 35058723 PMC8753526

[ref58] Srihera N. Li Y. Zhang T. T. Wang Y. M. Yanagita T. Waiprib Y. . (2022). Preparation and characterization of astaxanthin-loaded liposomes stabilized by sea cucumber sulfated sterols instead of cholesterol. J. Oleo Sci. 71, 401–410. doi: 10.5650/jos.ess21233 35153245

[ref59] Tian Z. R. Yao M. Zhou L. Y. Song Y. J. Ye J. Wang Y. J. . (2020). Effect of docosahexaenoic acid on the recovery of motor function in rats with spinal cord injury: a meta-analysis. Neural Regen. Res. 15, 537–547. doi: 10.4103/1673-5374.266065, PMID: 31571666 PMC6921345

[ref60] Tran A. P. Warren P. M. Silver J. (2018). The biology of regeneration failure and success after spinal cord injury. Physiol. Rev. 98, 881–917. doi: 10.1152/physrev.00017.2017, PMID: 29513146 PMC5966716

[ref61] Turck D. Castenmiller J. de Henauw S. Hirsch Ernst K. I. Kearney J. Maciuk A. . (2020). Safety of astaxanthin for its use as a novel food in food supplements. EFSA J. 18:e05993. doi: 10.2903/j.efsa.2020.5993, PMID: 32874213 PMC7448075

[ref62] Ueta T. Owen J. H. Sugioka Y. (1992). Effects of compression on physiologic integrity of the spinal cord, on circulation, and clinical status in four different directions of compression: posterior, anterior, circumferential, and lateral. Spine (Phila Pa 1976) 17, S217–S226. doi: 10.1097/00007632-199208001-00002, PMID: 1523504

[ref63] Wang Y. Branicky R. Noë A. Hekimi S. (2018). Superoxide dismutases: dual roles in controlling ROS damage and regulating ROS signaling. J. Cell Biol. 217, 1915–1928. doi: 10.1083/jcb.201708007, PMID: 29669742 PMC5987716

[ref64] Wang Z. He Z. Emara A. M. Gan X. Li H. (2019). Effects of malondialdehyde as a byproduct of lipid oxidation on protein oxidation in rabbit meat. Food Chem. 288, 405–412. doi: 10.1016/j.foodchem.2019.02.126, PMID: 30902311

[ref65] Wilcox J. T. Satkunendrarajah K. Nasirzadeh Y. Laliberte A. M. Lip A. Cadotte D. W. . (2017). Generating level-dependent models of cervical and thoracic spinal cord injury: exploring the interplay of neuroanatomy, physiology, and function. Neurobiol. Dis. 105, 194–212. doi: 10.1016/j.nbd.2017.05.009 28578003

[ref66] Xiang D. Jia B. Zhang B. Liang J. Hong Q. Wei H. . (2022). Astaxanthin supplementation improves the subsequent developmental competence of vitrified porcine zygotes. Frontiers in Veterinary Science. 9:871289. doi: 10.3389/fvets.2022.871289, PMID: 35433903 PMC9011099

[ref67] Xu J. Rong S. Gao H. Chen C. Yang W. Deng Q. . (2017). A combination of flaxseed oil and astaxanthin improves hepatic lipid accumulation and reduces oxidative stress in high fat-diet fed rats. Nutrients 9:271. doi: 10.3390/nu9030271, PMID: 28335388 PMC5372934

[ref68] Yao M. Yang L. Wang J. Sun Y. Dun R. Wang Y. . (2015). Neurological recovery and antioxidant effects of curcumin for spinal cord injury in the rat: a network Meta-analysis and systematic review. J. Neurotraum. 32, 381–391. doi: 10.1089/neu.2014.3520 25141070

[ref69] Yin Y. Han W. Cao Y. (2019). Association between activities of SOD, MDA and Na+-K+-ATPase in peripheral blood of patients with acute myocardial infarction and the complication of varying degrees of arrhythmia. Hell. J. Cardiol. 60, 366–371. doi: 10.1016/j.hjc.2018.04.003, PMID: 29702256

[ref70] Zhang X. Zhang X. Wu Q. Li W. Wang C. Xie G. . (2014). Astaxanthin offers neuroprotection and reduces neuroinflammation in experimental subarachnoid hemorrhage. J. Surg. Res. 192, 206–213. doi: 10.1016/j.jss.2014.05.029, PMID: 24948541

[ref71] Zhang X. Zhao X. Tie S. Wang H. Tan M. (2021). Ultrasonic self-emulsification nanocarriers for cellular enhanced astaxanthin delivery. J. Agric. Food Chem. 69, 2719–2728. doi: 10.1021/acs.jafc.0c05983, PMID: 33625837

[ref72] Zhao Y. Hu X. Liu Y. Dong S. Wen Z. He W. . (2017). ROS signaling under metabolic stress: cross-talk between AMPK and AKT pathway. Mol. Cancer 16:79. doi: 10.1186/s12943-017-0648-1 28407774 PMC5390360

[ref73] Zhou K. Zheng Z. Li Y. Han W. Zhang J. Mao Y. . (2020). TFE3, a potential therapeutic target for spinal cord injury via augmenting autophagy flux and alleviating ER stress. Theranostics. 10, 9280–9302. doi: 10.7150/thno.46566, PMID: 32802192 PMC7415792

[ref74] Zhou L. Chen X. Yu B. Pan M. Fang L. Li J. . (2022). The effect of metformin on ameliorating neurological function deficits and tissue damage in rats following spinal cord injury: a systematic review and network meta-analysis. Front Neurosci-Switz. 16:946879. doi: 10.3389/fnins.2022.946879, PMID: 36117612 PMC9479497

[ref75] Zhuang J. Zhou X. Liu T. Zhang S. Yuan F. Zhang L. . (2021). Astaxanthin attenuated hyperuricemia and kidney inflammation by inhibiting uric acid synthesis and the NF-κ B/NLRP3 signaling pathways in potassium oxonate and hypoxanthine-induced hyperuricemia mice. Pharmazie 76, 551–558. doi: 10.1691/ph.2021.1731, PMID: 34782040

[ref76] Zrzavy T. Schwaiger C. Wimmer I. Berger T. Bauer J. Butovsky O. . (2021). Acute and non-resolving inflammation associate with oxidative injury after human spinal cord injury. Brain 144, 144–161. doi: 10.1093/brain/awaa360, PMID: 33578421 PMC7880675

