# Supplementary material for: Astaxanthin promotes locomotor function recovery and attenuates tissue damage in rats following spinal cord injury: a systematic review and trial sequential analysis
Source: Front Neurosci. 2023 Oct 10;17:1255755. doi: 10.3389/fnins.2023.1255755 (PMC10595034; doi:10.3389/fnins.2023.1255755)
Supplement: Supplementary file 1 [file Data_Sheet_1.docx]

**Astaxanthin promotes locomotor function recovery and attenuates tissue damage in rats following spinal cord injury: a systematic review and trial sequence analysis**

**SUPPLEMENTARY MATERIAL**

**Table S1. Subgroup analyses of the effects of AST.**

| **Subgroup title** | **No. of studies** | **No. of animals** | **Weighted mean difference** | | **Heterogeneity** | | **Subgroup difference** |
| --- | --- | --- | --- | --- | --- | --- | --- |
|  |  |  | **95% CI** | ***P* value** | ***I*^2^** | ***P* value** |  |
| 1 Administration route | 7 | 100 |  |  |  |  |  |
| 1.1 BBB scores at day 3 | 4 | 58 |  |  |  |  | *P* = 0.69 |
| 1.1.1 Intrathecal | 2 | 34 | 2.90 [1.34, 4.46] | *P* = 0.0003 | 50 | *P* = 0.16 |  |
| 1.1.2 Intragastrical | 2 | 24 | 2.54 [1.65, 3.42] | *P* < 0.00001 | 56 | *P* = 0.13 |  |
| 1.2 BBB scores at day 7 | 6 | 88 |  |  |  |  | *P* = 0.02 |
| 1.2.1 Intrathecal | 4 | 64 | 3.73 [2.25, 5.21] | *P* < 0.00001 | 54 | *P* = 0.09 |  |
| 1.2.2 Intragastrical | 2 | 24 | 1.89 [1.41, 2.37] | *P* < 0.00001 | 0 | *P* = 0.95 |  |
| 1.3 BBB scores at day 14 | 6 | 88 |  |  |  |  | *P* = 0.10 |
| 1.3.1 Intrathecal | 4 | 64 | 4.84 [1.76, 7.93] | *P* = 0.002 | 90 | *P* < 0.00001 |  |
| 1.3.2 Intragastrical | 2 | 24 | 1.66 [-0.59, 3.92] | *P* = 0.15 | 93 | *P* = 0.0001 |  |
| 1.4 BBB scores at day 21 | 6 | 88 |  |  |  |  | *P* = 0.08 |
| 1.4.1 Intrathecal | 4 | 64 | 6.63 [2.79, 10.47] | *P* = 0.0007 | 94 | *P* < 0.00001 |  |
| 1.4.2 Intragastrical | 2 | 24 | 2.20 [-1.03, 5.44] | *P* = 0.18 | 96 | *P* < 0.00001 |  |
| 1.5 BBB scores at day 28 | 5 | 76 |  |  |  |  | *P* = 0.12 |
| 1.5.1 Intrathecal | 4 | 64 | 7.22 [3.37, 10.71] | *P* < 0.0001 | 91 | *P* < 0.00001 |  |
| 1.5.2 Intragastrical | 1 | 12 | 4.40 [3.82, 4.98] | *P* < 0.00001 |  |  |  |
| 2 Injury model | 6 | 88 |  |  |  |  |  |
| 2.1 BBB scores at day 7 | 6 | 88 |  |  |  |  | *P* < 0.0001 |
| 2.1.1 Contusion | 4 | 58 | 1.93 [1.48, 2.39] | *P* < 0.00001 | 0 | *P* = 0.74 |  |
| 2.1.2 Compression | 2 | 30 | 4.63 [3.41, 5.86] | *P* < 0.00001 | 0 | *P* = 0.51 |  |
| 2.2 BBB scores at day 14 | 6 | 88 |  |  |  |  | *P* < 0.00001 |
| 2.2.1 Contusion | 4 | 58 | 1.94 [0.76, 3.12] | *P* = 0.001 | 82 | *P* = 0.0009 |  |
| 2.2.2 Compression | 2 | 30 | 7.56 [5.68, 9.45] | *P* < 0.00001 | 0 | *P* = 0.94 |  |
| 2.3 BBB scores at day 21 | 6 | 88 |  |  |  |  | *P* < 0.00001 |
| 2.3.1 Contusion | 4 | 58 | 2.70 [1.13, 4.27] | *P* = 0.0007 | 88 | *P* < 0.0001 |  |
| 2.3.2 Compression | 2 | 30 | 10.25 [8.53, 11.97] | *P* < 0.00001 | 0 | *P* = 0.94 |  |
| 2.4 BBB scores at day 28 | 5 | 76 |  |  |  |  | *P* < 0.00001 |
| 2.4.1 Contusion | 3 | 46 | 4.32 [3.81, 4.83] | *P* < 0.00001 | 0 | *P* = 0.61 |  |
| 2.4.2 Compression | 2 | 30 | 10.54 [8.59, 12.50] | *P* < 0.00001 | 0 | *P* = 0.86 |  |

**Table S2. Summary of sensitivity analysis.**

| **BBB scale** | **Studies exclusion** | **No. of animals** | **Weighted mean difference** | | **Heterogeneity** | |
| --- | --- | --- | --- | --- | --- | --- |
|  |  |  | **95% CI** | ***P* value** | ***I*^2^** | ***P* value** |
| 1 BBB scale at 3^rd^ day | All included | 58 | 2.64 [1.99, 3.29] | < 0.00001 | 31 | 0.22 |
|  | Studies without outcome assessor blinding | 46 | 2.89 [2.31, 3.46] | < 0.00001 | 1 | 0.36 |
|  | Small sample studies | 34 | 2.90 [1.34, 4.46] | 0.003 | 50 | 0.16 |
|  | Mohaghegh et al., 2020 | 48 | 2.20 [0.84, 3.56] | < 0.00001 | 48 | 0.15 |
|  | Ren et al., 2019 | 46 | 2.92 [2.26, 3.58] | < 0.00001 | 37 | 0.21 |
|  | Masoudi et al., 2017 | 40 | 3.80 [2.05, 5.55] | < 0.00001 | 24 | 0.27 |
|  | Chen, 2014 | 46 | 2.00 [1.01, 2.99] | < 0.00001 | 1 | 0.36 |
| 2 BBB scale at 7^th^ day | All included | 88 | 2.85 [1.83, 3.87] | < 0.00001 | 72 | 0.003 |
|  | Studies without outcome assessor blinding | 64 | 2.87 [1.37, 4.36] | 0.0002 | 68 | 0.03 |
|  | Small sample studies | 52 | 3.47 [1.34, 5.60] | 0.001 | 63 | 0.07 |
|  | Abbaszadeh et al., 2023 | 76 | 4.36 [2.89, 5.83] | < 0.00001 | 58 | 0.05 |
|  | Li et al., 2021 | 76 | 1.90 [1.32, 2.48] | < 0.00001 | 73 | 0.005 |
|  | Mohaghegh et al., 2020 | 72 | 1.86 [0.02, 3.70] | < 0.00001 | 78 | 0.001 |
|  | Fakhri et al., 2019 | 70 | 5.26 [3.04, 7.48] | < 0.00001 | 63 | 0..03 |
|  | Masoudi et al., 2017 | 70 | 3.50 [0.71, 6.29] | < 0.00001 | 77 | 0.002 |
|  | Chen, 2014 | 76 | 1.87 [1.02, 2.72] | < 0.00001 | 76 | 0.002 |
| 3 BBB scale at 14^th^ day | All included | 88 | 3.46 [1.85, 5.07] | < 0.00001 | 90 | < 0.00001 |
|  | Studies without outcome assessor blinding | 64 | 3.30 [1.78, 4.81] | < 0.0001 | 81 | 0.001 |
|  | Small sample studies | 52 | 3.93 [0.91, 6.95] | 0.01 | 87 | 0.0004 |
|  | Abbaszadeh et al., 2023 | 76 | 7.50 [5.01, 9.99] | =0.0003 | 87 | < 0.00001 |
|  | Li et al., 2021 | 76 | 2.80 [2.05, 3.55] | 0.0008 | 91 | < 0.00001 |
|  | Mohaghegh et al., 2020 | 72 | 1.74 [0.88, 2.60] | 0.0003 | 92 | < 0.00001 |
|  | Fakhri et al., 2019 | 70 | 7.65 [4.76, 10.54] | 0.0003 | 89 | < 0.00001 |
|  | Masoudi et al., 2017 | 70 | 3.20 [1.09, 5.31] | 0.0001 | 92 | < 0.00001 |
|  | Chen, 2014 | 76 | 0.50 [-0.39, 1.39] | < 0.00001 | 87 | < 0.00001 |
| 4 BBB scale at 21^th^ day | All included | 88 | 4.96 [2.81, 7.11] | < 0.0001 | 94 | < 0.00001 |
|  | Studies without outcome assessor blinding | 64 | 4.81 [2.81, 6.82] | < 0.00001 | 90 | < 0.00001 |
|  | Small sample studies | 52 | 5.45 [1.59, 9.31] | 0.006 | 93 | < 0.00001 |
|  | Abbaszadeh et al., 2023 | 76 | 10.19 [7.83, 12.55] | < 0.0001 | 93 | < 0.00001 |
|  | Li et al., 2021 | 76 | 3.80 [3.33, 4.27] | 0.002 | 95 | < 0.00001 |
|  | Mohaghegh et al., 2020 | 72 | 2.57 [1.30, 3.84] | < 0.0001 | 95 | < 0.00001 |
|  | Fakhri et al., 2019 | 70 | 10.32 [7.82, 12.82] | 0.0001 | 93 | < 0.0001 |
|  | Masoudi et al., 2017 | 60 | 3.90 [2.23, 5.57] | < 0.0001 | 95 | < 0.00001 |
|  | Chen, 2014 | 76 | 0.50 [-0.75, 1.75] | < 0.00001 | 93 | < 0.00001 |
| 5 BBB scale at 28^th^ day | All included | 76 | 6.42 [4.29, 8.55] | < 0.00001 | 89 | 0.0001 |
|  | Studies without outcome assessor blinding | 64 | 5.46 [3.59, 7.32] | < 0.00001 | 85 | 0.0001 |
|  | Small sample studies | 52 | 6.19 [2.63, 9.75] | 0.0007 | 90 | < 0.0001 |
|  | Abbaszadeh et al., 2023 | 64 | 10.38 [7.66, 13.10] | < 0.0001 | 85 | 0.0001 |
|  | Li et al., 2021 | 64 | 4.40 [3.82, 4.98] | < 0.0001 | 91 | < 0.0001 |
|  | Mohaghegh et al., 2020 | 60 | 3.73 [2.42, 5.04] | < 0.00001 | 91 | < 0.00001 |
|  | Fakhri et al., 2019 | 58 | 10.72 [7.92, 13.52] | < 0.00001 | 85 | 0.0002 |
|  | Masoudi et al., 2017 | 58 | 4.70 [2.70, 6.70] | < 0.00001 | 92 | < 0.00001 |
